# To Be a Flower or Fruiting Branch: Insights Revealed by mRNA and Small RNA Transcriptomes from Different Cotton Developmental Stages

**DOI:** 10.1038/srep23212

**Published:** 2016-03-17

**Authors:** Quan Sun, Xiongming Du, Chaowei Cai, Lu Long, Sai Zhang, Peng Qiao, Weina Wang, Kexue Zhou, Guanghao Wang, Xin Liu, Hui Zhang, Shuaipeng Geng, Can Yang, Wei Gao, Jianchuan Mo, Chen Miao, Chunpeng Song, Yingfan Cai

**Affiliations:** 1 State Key Laboratory of Cotton Biology, Henan Key Laboratory of Plant Stress Biology, School of Life Sciences, School of Computer and Information Engineering, Henan University, Kaifeng 475004, China; 2 College of Bioinformation, Chongqing University of Posts and Telecommunications, Chongqing 400065, China; 3 State Key Laboratory of Cotton Biology, Cotton Institute of the Chinese Academy of Agricultural Sciences, Key Laboratory of Cotton Genetic Improvement, Ministry of Agriculture, Anyang, Henan 455000, China

## Abstract

The architecture of the cotton plant, including fruit branch formation and flowering pattern, is the most important characteristic that directly influences light exploitation, yield and cost of planting. Nulliplex branch is a useful phenotype to study cotton architecture. We used RNA sequencing to obtain mRNA and miRNA profiles from nulliplex- and normal-branch cotton at three developmental stages. The differentially expressed genes (DEGs) and miRNAs were identified that preferentially/specifically expressed in the pre-squaring stage, which is a key stage controlling the transition from vegetative to reproductive growth. The DEGs identified were primarily enriched in RNA, protein, and signalling categories in *Gossypium barbadense* and *Gossypium hirsutum*. Interestingly, during the pre-squaring stage, the DEGs were predominantly enriched in transcription factors in both *G. barbadense* and *G. hirsutum*, and these transcription factors were mainly involved in branching and flowering. Related miRNAs were also identified. The results showed that fruit branching in cotton is controlled by molecular pathways similar to those in *Arabidopsis* and that multiple regulated pathways may affect the development of floral buds. Our study showed that the development of fruit branches is closely related to flowering induction and provides insight into the molecular mechanisms of branch and flower development in cotton.

Cotton is one of the most important economic crops for natural textile fibre and oilseed and has been widely planted in the United States, China, India, Pakistan, Australia, Uzbekistan and the other countries^1^,^2^. Cotton architecture is primarily determined based on shoot branching patterns and flowering patterns that directly influence light exploitation, yield, planting area, the efficiency of harvest mechanisation and the cost of planting^2^,^3^,^4^,^5^. In cotton, the floral bud’s forming indicates the beginning of reproductive growth on the basis of prior vegetative growth, and most cultivated cotton varieties produce lateral branch from the leaf axils, then the lateral branch develops and differentiates into vegetative branches and fruit branches. Vegetative buds often arise from the lower leaf axils, around the first five nodes of the stem, whereas the fruiting branch arises from the upper leaf axils. Shortly after the pre-squaring stage, visible triangular buds (approximately 3 mm) appear on the axil. The main shoot and the vegetative branch display monopodial growth, but the fruit branches display sympodial growth, typically with more than two nodes^5^,^6^,^7^. Floral bud bursting and fruit branching are major events in the development of cotton architecture and are among the most important productivity-related agronomic traits considered in breeding and cultivation programs^8^,^9^.

The prominent characteristics of cotton are the extensive overlap between vegetative growth and reproductive growth and the requirement for manual/mechanical pruning or artificial chemical regulation to perform alterations of the cotton architecture, which are important determinants of cotton yield^10^,^11^. Thus, it is important to balance vegetative growth and reproductive growth by improving the architecture, flowering and branching pattern of cotton to obtain higher yields and a lower cost of planting.

In recent years, the molecular mechanisms involving plant architecture, including floral transition, branching, shoot apical meristem development, and branch angle formation, have been extensively studied in rice etc., including the central roles of Branched1 (BRC1) in strigolactones, branching and Tiller Angle Control 1 (TAC1) in plant architecture^12^,^13^,^14^,^15^,^16^. Previous studies have mapped quantitative trait loci (QTLs) of cotton architectural traits using molecular markers, including fruit branch internode length and fruit branch angle^8^,^9^,^17^,^18^,^19^. The molecular mechanisms involved in the development of cotton architecture traits have been rarely reported.

There is a type of cotton branching mutation called nulliplex branch, for which most of the flowers arise directly from the leaf axils on the main shoot, and these individuals typically do not have a fruiting branch^20^,^21^,^22^. There are 1–3 flowers on the leaf axils on the main shoot and the fruiting branches seem to disappear in this branch mutation cotton. A nulliplex branch is an atypical branch that is genetically determined and is considered a cluster branch trait^20^,^21^,^22^. The nulliplex branching trait is controlled by the recessive gene gb_nb1^5^. Cotton varieties with nulliplex branches have been planted in areas including Xinjiang, China, and Uzbekistan, and their unique plant architecture is suitable for high-density and mechanised planting, without needing pruning and chemical regulation, and with relatively short growing stages^5^. Chen *et al*. found that the nulliplex branch gene is localised to a 600 kb genome fragment and suggested that the trait may be related to an *Arabidopsis thaliana* centroradialis homologue (the ATC gene), which acts as a floral suppressor in *Arabidopsis*^5^,^23^. However, the molecular mechanisms of the development of nulliplex- and normal-branch cotton remain unknown.

The novel, high-throughput, deep-sequencing transcriptome approach known as RNA-seq has allowed for the generation of large-scale libraries of expressed sequence tags and has improved the speed of gene discovery in recent years. Beyond identifying and sequencing mRNA and small RNA transcriptomes, the Illumina RNA-seq platform allows researchers to examine the expression patterns of transcripts in tissues of interest. This approach has been successfully applied to a variety of plant species, including cotton^24^.

Small RNAs are short non-coding RNAs that play crucial roles in a wide range of biological processes, including cell proliferation, developmental timing, floral transition, and stress responses^25^,^26^,^27^,^28^. MicroRNAs (miRNAs) regulate a large number of genes in plants by binding to the 3′ UTR or other regions of target mRNAs leading to degradation or translational repression, which is common during development^29^. Previous studies in cotton have chiefly focused on miRNA expression in the ovules, during fibre development, and in the stress response^30^,^31^.

Recently, the genome of an allotetraploid cotton (*Gossypium hirsutum*) was published after the diploid species *Gossypium raimondii* and *Gossypium arboreum* were sequenced^1^,^32^,^33^,^34^. *G. hirsutum* is mainly cultivated as upland cotton, and the genomic data provides more comprehensive molecular information on production traits than genomic data for diploid species.

In this study, we performed high-throughput next-generation sequencing to identify the molecular mechanisms of the development of the fruit branch and floral bud in cotton. The square forming period of nulliplex and normal branches was used to simultaneously measure mRNA and miRNA expression profiles. Integration analysis of mRNA and miRNA expression and elucidation of the regulatory relationship between miRNA and their corresponding mRNA targets are crucial for understanding the development of fruit branch during the floral bud forming stage. Our results demonstrated that the differentially expressed genes (DEGs) between the nulliplex and normal branches in the pre-squaring stage of *G. barbadense* and *G. hirsutum* were primarily related to floral transition and branching development. These results provide a molecular basis for understanding the cotton plant’s architecture and imply that the development of the fruit branch may be associated with flowering transition.

## Results

### Transcriptome sequencing of mRNA libraries

**Figure 1:**
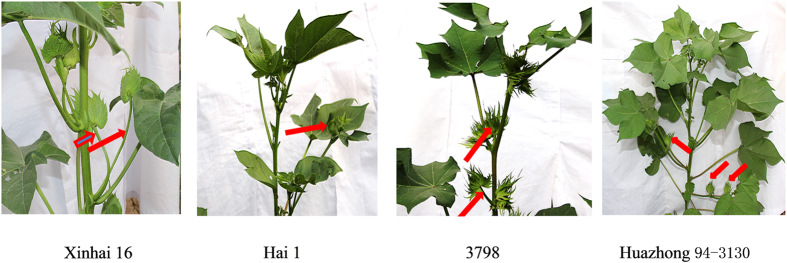
Plant appearance of bud on branch or main shoot in diffenrent cotton varieties (lines). Red arrows indicate bud on main shoot (Nulliplex-branch) or normal branch (wild type). Nulliplex-branch: 3798 (*G. hirsutum*) and Xinhai 16 (*G.barbadense*), normal branch(wild type):Huazhong94-3130 (*G. hirsutum*) and Hai1(*G.barbadense*).

To identify transcripts that are differentially expressed during the differentiation stage of flower and floral buds between nulliplex- and normal-branch cotton, samples from plants at three different developmental stages were used: the seedling stage, the pre-squaring stage, and the squaring stage. Four lines (varieties) were used to construct RNA-seq libraries (Fig. 1). The experiments were performed with three biological replicates, resulting in 36 RNA-seq libraries for sequencing. A total of 2,022,339,010 raw reads were obtained. After low-quality reads were filtered out, a total of 1,915,778,530 clean reads were selected for further analysis (See supplemental Table S1).

### DEGs between nulliplex- and normal-branch cotton

**Figure 2:**
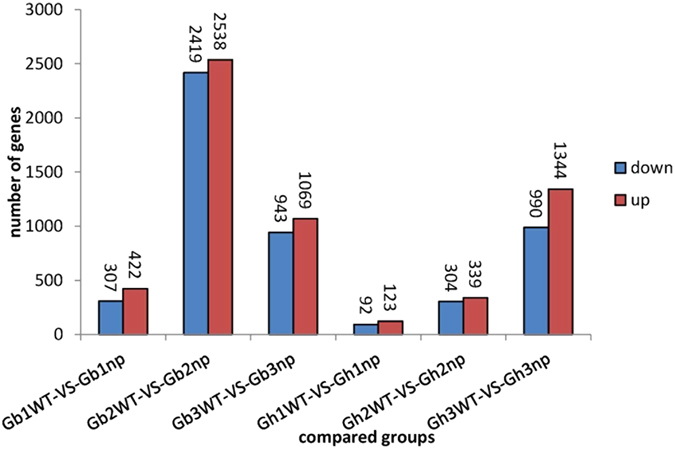
Numbers of different expressed genes in each comparison. The numbers on column showed quantity of up-regulated (red) and down-regulated (blue) genes.

Based on these expression analyses, we identified 422 upregulated and 307 downregulated DEGs in *G. barbadense* and 123 upregulated and 92 downregulated DEGs in *G. hirsutum* during the seedling stage. In *G. barbadense and G. hirsutum,* 2,538 upregulated and 2,419 downregulated and 339 upregulated and 304 downregulated DEGs, respectively, were identified during the pre-squaring stage and 1,069 upregulated and 943 downregulated and 1,344 upregulated and 990 downregulated DEGs, respectively, were identified during the squaring stage (Fig. 2, see supplemental Data set S1 to S6 online). There were more DEGs at the pre-squaring stage in *G. barbadense* than the other two stages, whereas the greatest number of DEGs was identified in the squaring stage in *G. hirsutum*.

### Functional category enrichment of DEGs

**Figure 3:**
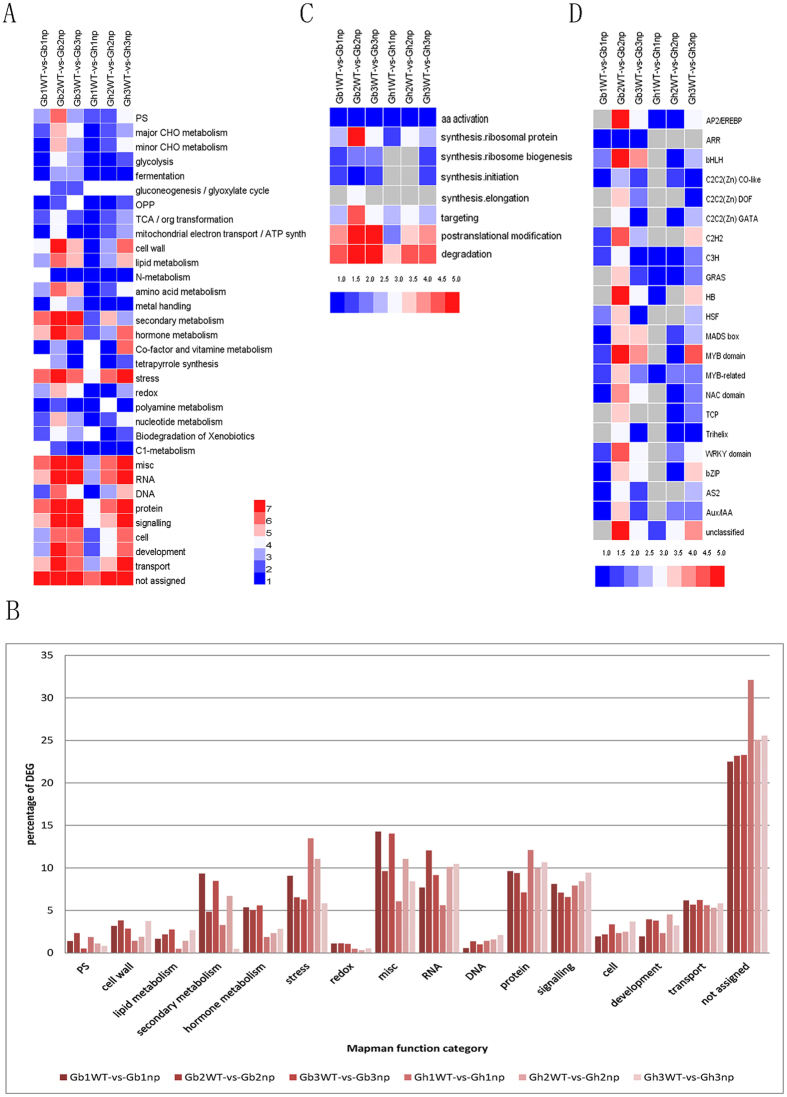
Enrichment of functional categories for DEGs in six compared groups. (**A**) Enrichment of functional categories for DEG lists. (**B**) Enrichment of protein subcategories. (**C**) Enrichment of transcription factor subcategories. (**D**) the percentage of each functional categories for DEGs in six compared groups. Gray, not significantly enriched. The bar showed the log2 value of enrich gene number.

All DEGs of the six groups were assigned to Mapman functional categories. During the seedling stage, the DEGs were almost equally distributed in each category. During the second and third stages, the DEGs were mainly enriched for RNA, protein, signalling, and transport in both types of cotton (Fig. 3A,B). Regarding protein metabolism categories, the DEGs were mainly associated with ribosomal protein, posttranslational modification, and degradation at the second stage of *G. barbadense*. The third stage was mainly enriched with genes for posttranslational modification and degradation. However, the DEGs were considerably enriched in genes regulating degradation at the second stage and enriched in genes involved in posttranslational modification and degradation at the third stage (Fig. 3C). Regarding RNA categories, the DEGs were mainly enriched for transcription factor families, including homeobox, MYB, AP2/EREBP, WRKY, C2H2, and bHLH at the second stage in *G. barbadense* and enriched for MYB at the squaring stage in *G. hirsutum* (Fig. 3D).

### DEGs in flower and floral bud development

**Figure 4:**
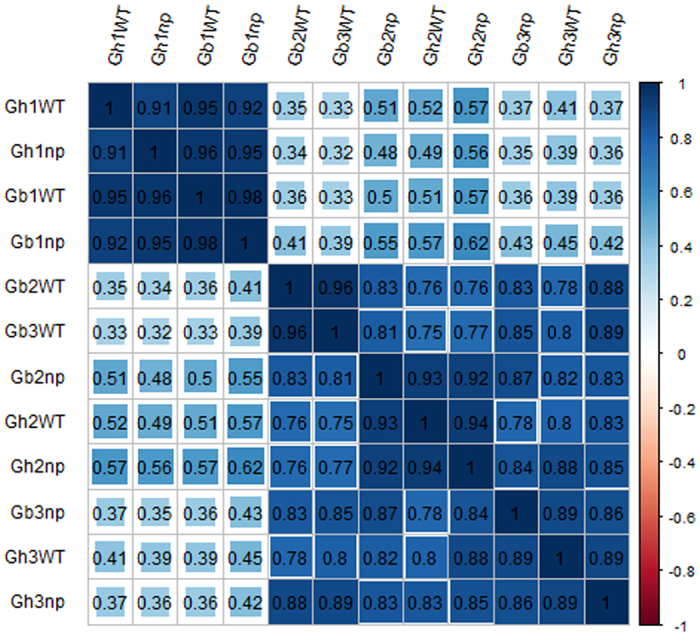
Correlation coefficients between gene expression data sets form three biological duplicates.

For interlibrary comparison, read numbers were normalised to relative abundance as reads per kilobase transcriptome per million mapped reads (RPKM). The RPKM value (mean value of three biological replicates) of each gene was further used to compute the related coefficients between each sample. The expression correlations of genes showed accordance between the same development stages in both types of cotton, excluding Gb3WT. The correlations were substantially lower between the seedling stage and squaring stage and/or pre-squaring stages (Fig. 4). Partial correlations between pre-squaring stage samples and squaring stage samples were lower than same-stage samples, such as that between Gb2WT and Gb2np with a coefficient of 0.83, which was slightly lower than 0.96 for Gb3WT (Fig. 4). This finding suggests that the development of the pre-squaring stage and squaring stage may be closely associated with integral processes.

**Figure 5:**
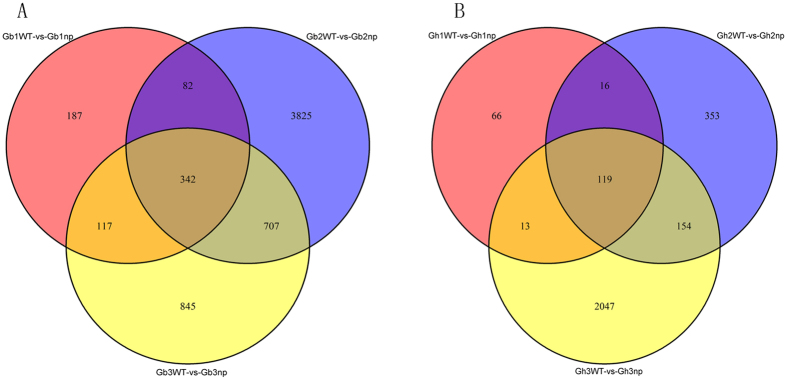
Venn diagram analyses of stage-specific expression genes in *G.barbadense* (**A**) *G. hirsutum* (**B**).

To study the molecular mechanisms of cotton fruit branch initiation and flower development, we analysed the stage-specific DEGs at three stages in each type of cotton. There were 187, 3,825, and 845 DEGs in the seedling stage, pre-squaring stages and squaring stages of *G. barbadense* between nulliplex and normal branches, respectively (Fig. 5A). In *G. hirsutum*, there were 66, 353, and 2,047 specific DEGs in the three stages between the branching types, respectively (Fig. 5B).

### DEGs specifically expressed in the pre-squaring stage

**Figure 6:**
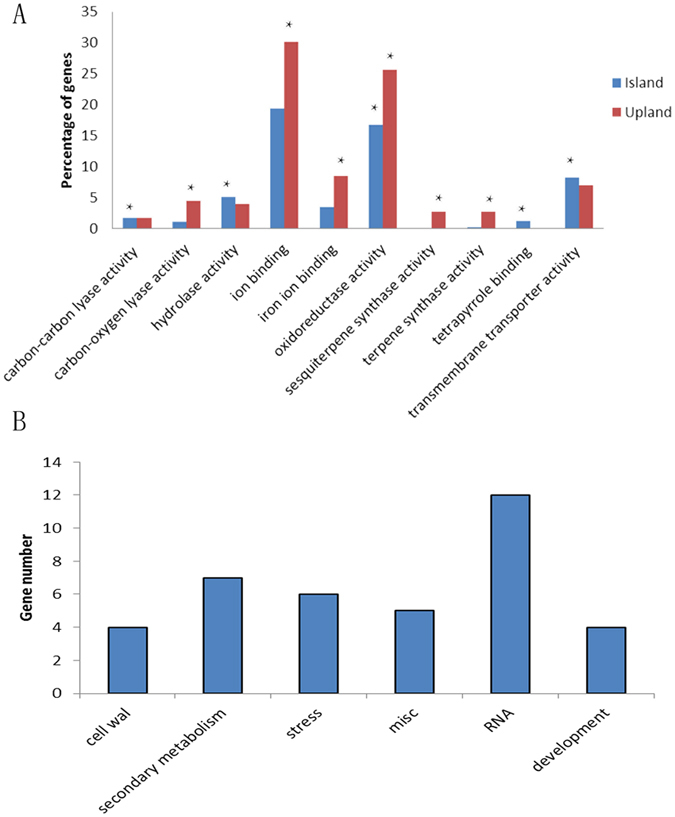
Enrichment of GO and Mapman functional categories for stage specific DEG in square initiating stage. (**A**) Go enrichment of the DEG between nulliplex branch and normal branch in *G. hirsutum* and *G.barbadense* in square initiating stage. (**B**) Enrichment of functional categories for DEG both in *G. hirsutum* and *G.barbadense*. Asterisk indicates *p* < 0.05.

Flowers and floral buds are forming in the pre-squaring stage; genes specifically expressed during this stage may play a key role in the early regulation of cotton flower and floral bud formation. Gene ontology (GO) enrichment analyses of DEGs specifically expressed during the pre-squaring stage revealed a major enrichment of oxidoreductase activity in both *G. barbadense* and *G. hirsutum* (see supplemental Tables S2 and S3; correction *p* < 0.05). In addition, DEGs were primarily enriched for transmembrane transporter activity and hydrolase activity and acting on glycosyl bonds in *G. barbadense* and ion binding in *G. hirsutum* (Fig. 6A, Tables 1 and 2). We further analysed the DEGs collectively expressed in the pre-squaring stage, and the results revealed 68 DEG candidates (See supplemental Fig. S1 and S2).

Then, we classified the genes that were specifically expressed during the pre-squaring stage using the Mapman program and found that these genes fell into six major groups: cell wall-related processes, secondary processes, stress response, miscellaneous, RNA processes, and development (Fig. 6B). More interestingly, 12 DEGs (approximately one-fifth of the tissue-specific genes) in the pre-squaring stage were mainly enriched in RNA processes, including key flower development genes such as short vegetative phase (SVP, CotAD_47245), late elongated hypocotyl (LHY, CotAD_32839), and pseudo-response regulator 5 (PRR5, CotAD_14148), and were related to ABI3/VP1 1 (RAV1, CotAD_22031), suggesting that the pre-squaring stage specifically expressed genes that tend to promote flower development. In addition, genes specifically expressed during bud and shoot apical meristem development included branched 1 (BRC1, CotAD_02759), breast cancer associated RING 1 (BARD1, CotAD_54816), and indole-3-acetic acid inducible 18 (IAA18, CotAD_00235), which implied that these genes control the growth and development of floral buds to form the cotton architecture (Table 3).

**Figure 7:**
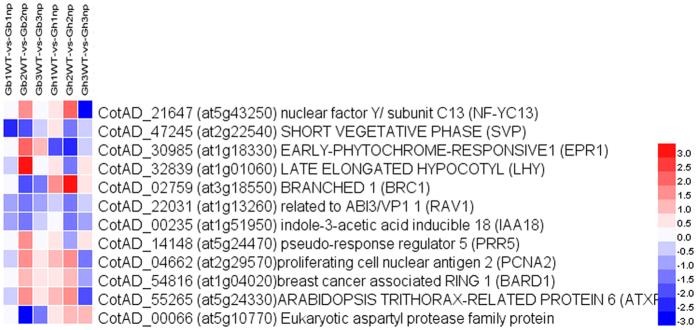
Expression profiles for transcription factors in square initiating stage-specific DEGs both in upland and *G.barbadense*. The bar showed log2 value of fold change.

SVP, RAV1, and IAA18 were substantially downregulated and NF-YC13, PCNA2, BARD1, and ATXRP were upregulated during the pre-squaring stage compared to the other two stages in both *G. barbadense* and *G. hirsutum*. Moreover, EPR1, PPR5, and LHY were upregulated in *G. barbadense* but downregulated in *G. hirsutum* at the second stage. For BRC1 and CotAD_00066, the tendency to change was in the opposite direction of the latter three genes (Fig. 7, see supplemental Fig. S2).

### DEGs of different developmental stages

**Figure 8:**
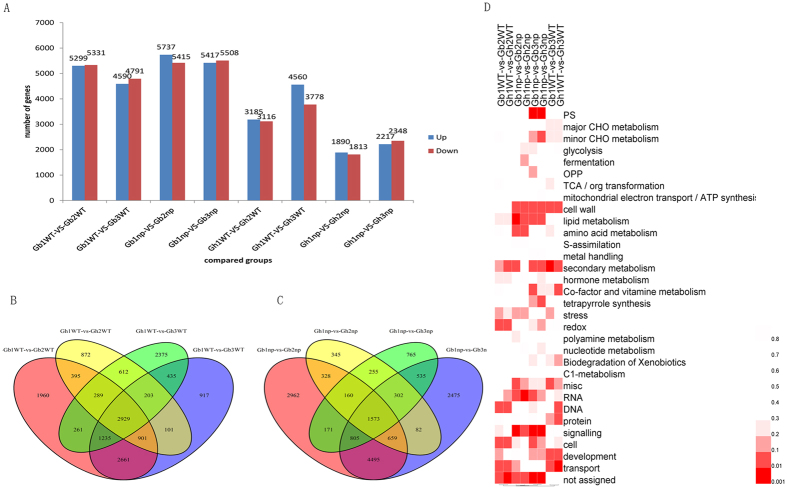
The distribution of DEG in different development stages. (**A**) Numbers of different expressed genes in each comparison. The numbers on column showed quantity of up-regulated (red) and down-regulated (blue) genes. (**B**,**C**) Venn diagram analyses of different stages in normal branch cotton and nulliplex branch cotton. (**D**) Enrichment of function categories for DEG. Gray, not significantly enriched. The bar mean the *p* value, *p* < 0.05 were significantly enriched.

The DEGs were analysed among the different developmental stages of each cotton variety, and the results showed that the number of DEGs in the squaring stage compared to the seedling stage and pre-squaring stage compared to seedlings in *G. barbadense* was much higher than in *G. hirsutum* in both nulliplex- and normal-branch cotton (Fig. 8A). The pre-squaring stage may be controlled by the differentiation of the fruit branch tissue, whereas the squaring stage is mainly controlled by the growth of the fruit branches in normal-branch cotton. The results revealed 395 DEGs between the pre-squaring stage and seedling stage and 435 DEGs between the squaring stage and seedling stage in normal-branch cotton (Fig. 8B). In nulliplex branches, there were 328 DEGs between the pre-squaring stage and the seedling stage and 535 DEGs between the squaring stage and the seedling stage.

These DEGs were enriched for functional categories, as determined using the Mapman software. The results showed that the DEGs of the nulliplex branches (Gb1np-vs-Gb2np, Gb1np-vs-Gb3np, Gh1np-vs-Gh2np, and Gh1np-vs-Gh3np) were substantially enriched in signalling categories, and the DEGs of the normal branches (Gb1WT-vs-Gb2WT, Gb1WT-vs-Gb3WT, Gh1WT-vs-Gh2WT, and Gh1WT-vs-Gh3WT) were mainly enriched in transport categories (Fig. 8D). In addition, the DEGs of the normal branches in the floral bud’s forming stage and seedling stage (Gb1WT-vs-Gb3WT and Gh1WT-vs-Gh3WT) were considerably enriched in development categories. These results suggest that nulliplex-branch and normal-branch cotton may have different developmental patterns.

### Sequencing of small RNA libraries

Twelve small RNA samples (each sample was the same as above with three biological repeats) yielded a total of 422,920,856 reads and 175,305,470 unique reads. There were more than 11 million reads and more than 4 million unique reads for each library. The reads of each library were mapped back to the cotton genome and accounted for 75% to 88% of the total reads, representing 67% to 81% of their unique read counterparts (See supplemental Table S4). In both types of cotton, rRNA in the squaring stage were substantially different between nulliplex and normal branches, showing 0.56% unique reads in normal branches and 1.36% unique reads in nulliplex branches for *G. barbadense* (miGb3WT vs. miGb3np) and 0.38% unique reads in the normal branches and 0.85% unique reads in nulliplex branches for *G. hirsutum* (miGh3WT vs. miGh3np). However, the other RNA families had similar proportions, including miRNA (0.5–0.7% for unique reads), snRNA (0.02–0.05% for unique reads), snoRNA (approximately 0.02% for unique reads), and tRNA (approximately 0.15% for unique reads) (See supplemental Table S4). For all sRNA libraries, the major distribution of read lengths was between 21 and 24 nt.

### Identification of known conserved miRNA families

A total of 712 known conserved miRNAs were identified in 12 samples (three biological replications). Of these, some miRNAs were highly and stably expressed in all samples, such as miR157a-5p, miR166a-3p, and miR3954 (See supplemental data set S7 online). However, other miRNA counts diverged sharply. For example, miRNA167h had more than 35 k reads in miGb3WT and miGb2np and no reads in miGb3np and miGh2np (See supplemental data set S7 online).

### Differentially expressed miRNA (DEM) in three stages of cotton

**Figure 9:**
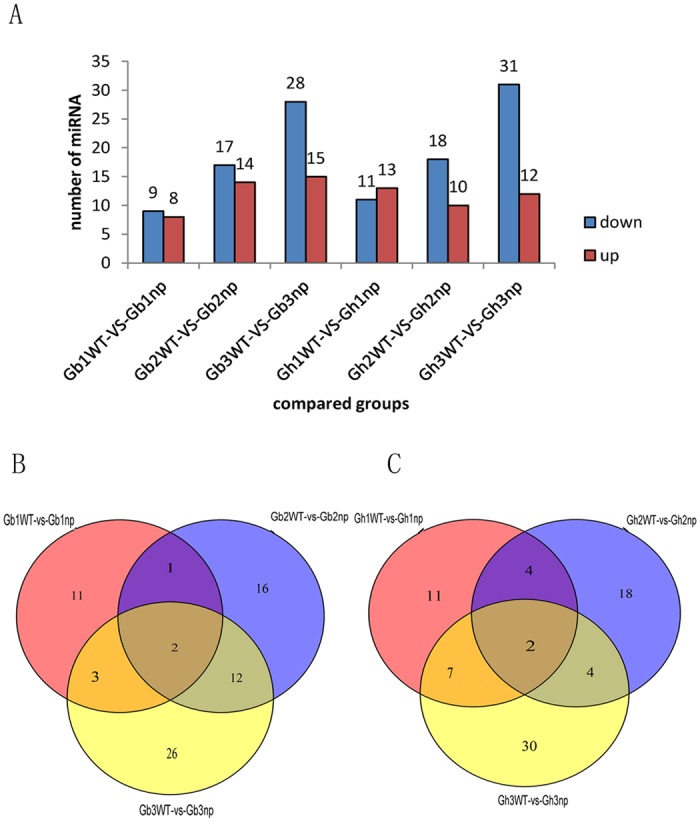
The statistics of differentially expressed miRNA. (**A**) Numbers of different expressed miRNA in each comparison. The numbers on column showed quantity of up-regulated (red) and down-regulated (blue) genes. Venn diagram analyses of stage-specific expression miRNAs in *G.barbadense* (**B**) *G. hirsutum* (**C**).

To study the regulation of miRNA in development of floral buds and fruit branch in cotton, we further analysed conserved DEMs using similar strategies as that used for RNA-seq. There were 17 (9 downregulated and 8 upregulated), 31 (17 downregulated and 14 upregulated), and 43 (28 downregulated and 15 upregulated) DEMs in the seedling stage, pre-squaring stage, and squaring stage of *G. barbadense* between nulliplex and normal branches, respectively (Fig. 9A, See supplemental data set 8–10 online). In *G. hirsutum*, there were 24 (11 downregulated and 13 upregulated), 28 (18 downregulated and 10 upregulated), and 43 (31 downregulated and 12 upregulated) DEMs, respectively (Fig. 9A, See supplemental data set S11–S13 online).

Among these DEMs, there were 11, 16, and 26 stage-specific DEMs in the *G. barbadense* seedling stage, pre-squaring stage, and squaring stage, respectively (Fig. 9B). In *G. hirsutum*, we identified 11, 18, and 30 stage-specific DEMs in the seedling stage, pre-squaring stage, and squaring stage, respectively (Fig. 9C). In *G. barbadense*, the uniquely expressed miRNAs at the pre-squaring stage, which included miR156c-3p, miR156i-3p, and miR171c of the miR156 and miR171 family may be related to floral transition (See supplemental Table S5)^27^. In *G. hirsutum*, the specifically expressed miRNAs at the pre-squaring stage including miR166c, a member of the miR166 family, were also involved in floral organ polarity and shoot apical meristem formation (See supplemental Table S6)^27^,^35^. In addition, at the pre-squaring stage of *G. barbadense*, the DEMs included miR167 and miR166 family members and the stage-specific DEMs noted above (See supplemental data set S9 online).

Novel miRNAs were identified and the results showed that of these 3,350 novel miRNAs, 13, 8, and 13 were DEMs in the seedling stage, pre-squaring stage, and squaring stage of *G. barbadense* and 11, 9, and 13 were DEMs in the three stages of *G. hirsutum*, respectively (See supplemental Fig. S3A and S3B).

### DEM target identification at three stages

The DEM target pairs above were identified from *G. hirsutum* genome sequences based on negative regulation between miRNA and their targets by combining the results of DEG analysis with RNA-seq. Among the three stages, 80 targets of the specifically expressed miRNAs were screened out. In the pre-squaring stage of *G. barbadense*, 11 DEMs targeting 32 DEGs were identified, including CotAD_35367 (MADS box protein, a homologue of sepallata3 (SEP3), which is involved in flower development)^36^ and CotAD_75643 (a homologue of HB8, which regulates post-embryonic meristem initiation and is also bound by SVP in vegetative tissue in *Arabidopsis*) (Table 4)^37^,^38^.

In the seedling stage, there were two DEMs with three target DEGs in *G. barbadense* and one DEM and one target in *G. hirsutum*. At the squaring stage, 7 DEMs were found in 11 DEG target genes in *G. barbadense*, and in *G. hirsutum*, 6 DEMs were identified with a predicted 33 target genes (Table 4).

### RNA-seq expression validation by qPCR

We used qPCR to confirm the reliability of the RNA-seq data. The results showed that among nine selected DEGs of the pre-squaring stages, approximately 90% were consistent with RNA-seq data (See supplemental Fig. S5).

## Discussion

Plant architecture is fundamental to agricultural productivity and the artificial selection of desired growth habits. The regulation of cotton architecture has enormous potential applications for high-density planting, mechanised harvesting, decreasing the cost of cultivation and improving yield. In the present study, to investigate the intricate molecular mechanisms of fruit branch and flower development in cotton plants, RNA-seq technology was utilized to examine the global gene expression profile of shoot apexes by generating both mRNA and miRNA libraries from the shoot apex of four cotton varieties (lines), including two with nulliplex branches and two with normal branches in three developmental stages with the aim of subtracting the unrelated differences in genotype, applying the newly published allotetraploid genome of *G. hirsutum* as a reference genome, which provided us with more complete and informative results for credible data mining^33^,^34^.

While it would have been highly desirable to start with genetic material that differs only in the trait of interest (e.g., near-isogenic lines), it would have taken many years to establish such lines given that *G. hirsutum* and *G. barbadense* are allotetraploid.

In this study although the nulliplex branches and normal branches lines of *G. hirsutum* and *G. barbadense* did not share parentage with each other, the differentially expressed genes observed should include the result of both the np mutation and unrelated differences in genotype in each comparison of *G. hirsutum* or *G. barbadense*. In order to acquire the result of the np mutation and subtract the unrelated differences in genotype, our experimental design with three different developmental stages of the two cotton fruit branch types in *G. hirsutum* and *G. barbadense* can help to find the differentially expressed genes related to the development of fruit branch. Firstly, we screened the common DEGs among three stages (the seedling, pre-squaring and squaring stage) in two kinds of branch type (nulliplex-branch and normal branch) of *G. barbadense* and *G. hirsutum* lines, respectively. Secondly, the common DEGs between the above two groups of *G. barbadense* and *G. hirsutum* were selected and can be considered as candidate DEGs for the np mutation. Finally, in this way we have in reality obtained interesting results and selected 68 genes associated with development of the np mutation in the pre-squaring stage. Among them, we have acquired the key genes related to development of branch (Table 3), such as Branched1(*GhBRC1*), IAA 18 etc., which play central roles in the development of branch in plants^13^,^39^,^40^,^41^,^42^,^43^,^44^. The results showed that the nulliplex branch is a useful trait and model for the study of cotton architecture.

The results indicated that the number of DEGs was significantly higher in the second stage than in the third stage of development in *G. barbadense*, but the DEG numbers of two stages in *G. hirsutum* showed the opposite results. The correlation coefficient between Gh2np and Gh2WT was 0.93, significantly higher than the 0.89 correlation found between Gh3np and Gh3WT. The correlation coefficient between Gb2np and Gb2WT was 0.83, lower than the correlation coefficient of 0.84 between Gb3np and Gb3WT (Fig. 4).

Mapman software analysis indicated that each developmental stage of the two cotton plant types was enriched with DEGs. The four samples of the seedling stage were still in the early period of vegetative growth, and many biochemical pathways were not initiated; consequently there was a relatively small number of DEGs. The pre-squaring stage is a key stage that may control the transition from vegetative growth to reproductive growth, and both types of growth may occur simultaneously during the squaring stage. DEGs between nulliplex branches and normal branches were mainly enriched in RNA, protein, secondary metabolism, stress, and signalling processes in the pre-squaring stage and squaring stage (Fig. 3A). The results showed remarkable differences between nulliplex-branch and normal-branch cotton in the development of the shoot apex, especially in the RNA category, which contains a large number of transcription factors. Fruiting branch development and differentiation from the apical or lateral meristem may be regulated by a series of transcription factors in cotton. However, there were different proportions of DEGs in some functional categories between *G. barbadense* and *G. hirsutum* (Fig. 3B). In the hormone metabolism category, the proportion of DEGs was significantly higher in *G. barbadense* than in *G. hirsutum*. This difference indicates that the developmental process may be more closely regulated by plant hormones in *G. barbadense* than in *G. hirsutum* in these four varieties (lines).

Regarding the different stages, the DEGs of the normal-branch cotton plants in the squaring stage and seedling stage (Gb1WT-vs-Gb3WT and Gh1WT-vs-Gh3WT) were remarkably enriched in development categories. In normal-branch cotton, the fruit branch grows during the squaring stage (there are no fruit branches in nulliplex-branch cotton), which is in agreement with the RNA-seq results. The second stage is a key developmental stage that regulates the transition from vegetative growth to reproductive growth and fruit branch formation. We further examined specific-stage DEGs in the second stage, and 68 candidate genes were identified.

The screened DEGs included 12 transcription factors (Table 3). The gene CotAD_02759 (*GhBRC1*), a homologue of *Arabidopsis* branched1 (*AtBRC1*), was downregulated in nulliplex-branch *G. barbadense* and upregulated in nulliplex-branch *G. hirsutum* at the pre-squaring stage. BRC1 downregulation leads to branch outgrowth in *Arabidopsis*^39^. Based on mutants and expression analysis in *Arabidopsis*, BRC1 is likely downstream of the more axillary growth pathway and is required for auxin-induced apical dominance^13^,^39^,^40^. The cotton *GhBRC1* gene may have the same function as *AtBRC1*, and the normal branch (fruit branch) is analogous to branch outgrowth in *Arabidopsis* to form axillary meristems. Cotton *GhBRC1* was upregulated in Gh2np (nulliplex-branch, *G. hirsutum*), which may be repressed during fruit branch development. However, it remains unclear why GhBRC1 was downregulated in Gb2np, a nulliplex-branch (no fruit branch) variety of *G. barbadense* (Fig. 6). The homologue (CotAD_00235, *GhIAA18*) of the auxin-related transcription factor IAA18 (indole-3-acetic acid inducible 18) in *Arabidopsis,* which causes aberrant cotyledon placement in embryos apical patterning, has been shown to be downregulated in nulliplex-branch cotton of both *G. barbadense* and *G. hirsutum*^41^. This finding suggests that another regulated pathway of lateral development may play a role.

The axillary meristem differentiates into fruit branches to form floral buds. Based on our results, the homologous genes may also regulate the differentiation of apical meristems in addition to BRC1 modulation of florigen activity in the floral buds of the axillary meristems in *Arabidopsis*^13^. The gene CotAD_54816 (*GhBARD1*) is homologous with BARD1 in *Arabidopsis* (also named ROW1) and is a repressor of Wuschel1^45^. BARD1 regulates shoot apical meristem organisation and maintenance by limiting WUS expression to the organising centre. Recently, BARD1 was shown to be essential for QC maintenance and stem cell niche development through the repression of WOX5 in the proximal meristem^45^,^46^. During the pre-squaring stage, *GhBARD1* was substantially upregulated in Gb2np and Gh2np, which may repress downstream gene expression or the shoot apical meristem formation of floral buds, causing nulliplex-branch cotton.

In addition, *GhBRC1* and *GhBARD1* are involved in floral meristem determinacy. BRC1 interacts with Flowering Locus T to repress the floral transition, and BARD1 can limit WUS expression, which forms a negative feedback loop with agamous (AG) and is involved in floral determinacy and the specialisation of reproductive floral organs^13^,^47^,^48^,^49^. These results suggest that the formation of nulliplex-branch cotton is associated with lateral axillary meristem development and floral formations, similar to other flowering time genes.

Interestingly, in addition to *GhBRC1* and *GhBARD1*, which may be involved in the flowering time network, there are four other *Arabidopsis* homologous genes among 12 transcription factors that may be related to the flowering time network, including CotAD_47245 (*GhSVP*, a homologue of short vegetative phase [SVP]), CotAD_32839 (*GhLHY*, a homologue of late elongated hypocotyl [LHY]), CotAD_30985 (*GhEPR1*, a homologue of early-phytochrome-responsive1 [EPR1), and CotAD_14148 (*GhPPR5*, a homologue of pseudo-response regulator 5 [PRR5]) (Table 3, Fig. 7).

In *Arabidopsis*, PRR5 can bind to LHY promoters and recruit transcriptional corepressors of the Groucho/tup1 corepressor family to repress LHY transcription in circadian clocks^50^,^51^. EPR1 is regulated by both phytochrome A and phytochrome B and is a component of a slave circadian oscillator in *Arabidopsis*^52^. To the best of our knowledge, the circadian clock gene and photoperiodic response can induce early flowering in *Arabidopsis*^53^,^54^,^55^. Based on our results, *GhPRR5, GhLHY*, and *GhEPR1* genes were upregulated in Gb2np and downregulated in Gh2np (Fig. 7).The same expression trend implied that *GhPRR5* might also bind to the *GhLHY* promoter and regulate the circadian clock of flowering time in cotton.

In addition, SVP is a negative regulator of the floral transition, which forms a flowering repressor complex together with FLC in *Arabidopsis*^56^,^57^,^58^,^59^. In our study, the SVP homologue *GhSVP* was downregulated in nulliplex-branch plants in both *G. barbadense* and *G. hirsutum*, which suggests that *GhSVP* may promote floral transition in nulliplex-branch cotton compared to normal-branch cotton in our four varieties (lines). Furthermore, the overexpression of RAV1, a homologue of cotton CotAD_22031 (*GhRAV1*) in *Arabidopsis*, slows the development of lateral roots and rosette leaves^60^,^61^,^62^. In addition, low expression causes an early flowering phenotype, implying that RAV1 may function as a negative regulator of growth and development^60^. In the present study, the *GhRAV1* gene was downregulated in nulliplex-branch cotton at the pre-squaring stage in both *G. barbadense* and *G. hirsutum*, which implies that *GhRAV1* may have accelerated the flowering speed of cotton varieties Xinhai16 and 3798.

Although there is no report on trehalose-6-phosphate synthase 5 (TPS5: the homology of cotton gene CotAD_16567, *GhTPS5*), TPS1 (a homology family number with TPS5) is essential for normal vegetative growth and transition to flowering in *Arabidopsis*^63^.

Similarity analyses of the above differentially expressed genes showed that many DEGs of the fruit branch and floral transition may be predominantly expressed in cotton similar to *Arabidopsis, Theobroma cacao, Populus euphratica*, and other species. However, most of the related evidence has been obtained from studies on *Arabidopsis*.

In the past decade, numerous studies have demonstrated that different miRNA families play important roles in regulating plant architecture and branching patterns^42^,^64^,^65^, although a high level of conservation of miRNAs and their regulatory pathways/target genes has been demonstrated in many species. In the present study, DEMs were analysed between nulliplex-branch and normal-branch plants. In the pre-squaring stage, stage-specific DEMs such as miR156 and miR171 family members were found in *G. barbadense* (See supplemental table S5) and miR166 was found in *G. hirsutum* (See supplemental table S6). The miR156 miRNA family promotes juvenile-to-adult phase transition and apical meristem formation^64^,^66^, and miR171 negatively regulates shoot branching^35^,^67^. In the pre-squaring stage, the miR166 family found in *G. hirsutum* regulates the shoot apical meristem and lateral organ formation in *Arabidopsis*^66^,^68^. In addition, these miRNA families have been confirmed to be closely related to flowering time^26^,^60^, in agreement with our RNA-seq results.

The majority of the miRNA families are expressed during several plant growth periods or developmental processes. The non-stage-specific DEMs may also be involved in branching and flowering processes. More members of mi156, miR166, and miR167 were differentially expressed in three developmental stages (See supplemental data set S8 to S13 online). Nevertheless, the target of the DEMs was predicted, and we could not identify the genes related to branching and flowering (Table 4). These results imply that there were a number of genes involved in branching and flowering that had not been previously identified, but the molecular mechanism of regulation remains unclear. This conclusion suggests that nulliplex branching may be related to a flower time-related candidate gene^5^.

In nulliplex-branch cotton, the floral bud directly arises from the leaf axils of the main shoot, whereas in normal-branch the floral bud arises from the node of the fruit branch. With the fruit branch growing on leaf axils, the floral bud is produced at the same time. Based on the above results, genes controlling the formation of nulliplex-branch cotton may also be involved in floral induction. Interestingly, in the four varieties (lines) used for sequencing in this study, the squaring stage of Xinhai16 (*G. barbadense* L., nulliplex branch) was significantly earlier than that of Hai1 (*G. barbadense* L., normal branch) and line 3,798 (*G. hirsutum* L., nulliplex-branch) was significantly earlier than Huazhong 94-3130 (*G. hirsutum* L., normal branch). The earlier squaring stage (flowering time) in the nulliplex-branch compared to the normal-branch cotton in both *G. barbadense* and *G. hirsutum* in the four varieties (lines) may coincide with branching control genes involved in floral induction.

**Figure 10:**
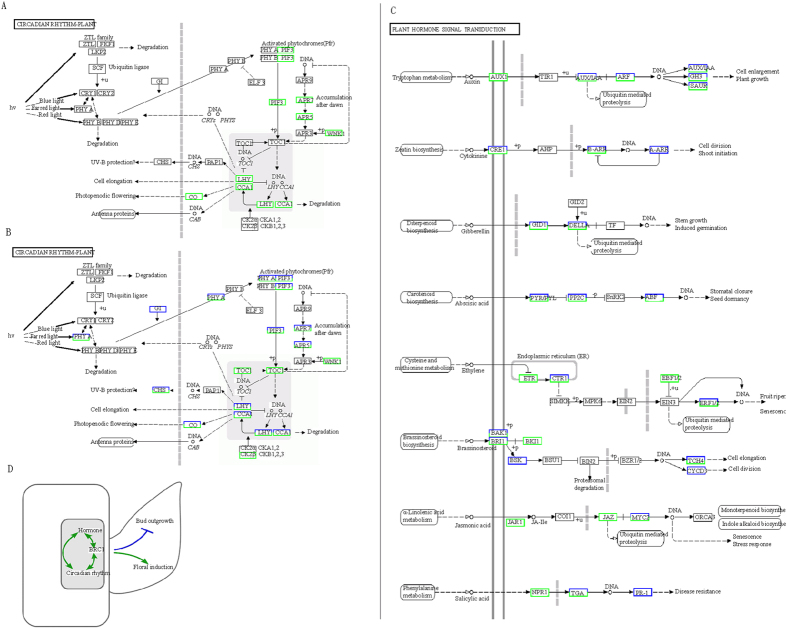
Schematic diagram in KEGG pathway of DEGs in square initiating stage by RNA-Seq data analysis and the model of bud outgrowth. (**A**) and (**B**) DEGs enriched in circadian rhythm pathway of *G. hirsutum* and *G.barbadense*, respectively. (**C**) DEGs enriched of plant hormone signal transduction pathway in G.barbadense. Up-regulated genes are colored red, down-regulated genes are colored green. (**D**) Proposed model for cotton fruit branch.

Our results showed that multiple pathways, including genes related to fruit branch development, e.g., GhBRC1, may control nulliplex branching. At the same time, the flowering time genes may influence fruit branch growth. The different tendencies of DEGs in the island and *G. hirsutum* imply that multiple pathways may regulate branching. The KEGG pathway of the pre-squaring stage DEGs showed that the DEGs of the island and *G. hirsutum* were both enriched in circadian rhythm genes, which may be associated with flowering (See supplemental Tables S5 and S6). In the circadian rhythm pathway, all enriched DEGs were downregulated (Fig. 10A). However, DEGs such as *GhLHY* have different expression patterns in *G. barbadense* compared to *G. hirsutum*. Other DEGs homologous to *GhLHY* showed the same expression trend as *GhLHY* in *G. hirsutum*, such as CotAD_17426. In addition, other genes in the circadian rhythm pathway have the same condition, such as CCA1, PIF3, and APR7 (Fig. 10B). Furthermore, the plant hormone signal transduction pathway was substantially enriched in *G. barbadense* but not in *G. hirsutum* (See supplemental Tables S5–S7). This finding supports the hypothesis that other regulation pathways may be involved in *G. barbadense*, and the results showed that many DEGs were related to multi-hormone signal transduction pathways (Fig. 10C, See supplemental Table S7).

In conclusion, *GhBRC1* may play a key role in floral bud formation (flowering) and fruit branch development in cotton, which is similar to BRC1′s central role in the architecture of *Arabidopsis*^42^,^43^. *GhBRC1* may also be associated with plant hormones and the flowering network, including the circadian rhythm genes that control fruit branching and floral transition, and thus is involved in elaborate regulatory mechanisms and networks.

## Conclusions

Overall, our results imply that the molecular pathways similar to those in *Arabidopsis* may control fruit branching in cotton. Multiple regulated pathways may affect the development of floral buds on leaf axils in *G. barbadense* and *G. hirsutum*. The development of the fruit branch is closely related to the induction of flowering. Our results provide a basis for plant type research in crops and an approach for elucidating the molecular mechanism of the development of the nulliplex branch and the normal branch in the flowering and fruit branch forming stage for improving plant architecture and yield in cotton and other plants.

## Materials and Methods

### Plant material

The cotton varieties Xinhai16 (*G. barbadense* L., nulliplex-branch), Hai1 (*G. barbadense* L., normal branch), line 3798 (*G. hirsutum* L., nulliplex-branch), and line Huazhong 94-3130 (*G. hirsutum* L., normal branch, obtained from the Cotton Institute of the Chinese Academy of Agricultural Sciences of China) were grown in a natural field environment (Kaifeng, China) in 2014, all the materials are stable and pure lines or varieties which have been self-fertilized for at least eight generations before we used in the studies. Shoot apices (approximately 10 mm) from each individual were collected at three sampling stage and stored immediately in liquid nitrogen after washing with water (See supplemental Fig. 3). The three stages used for constructing RNA-seq libraries included the seedling stage with two leaves of Xinhai16 (Gb1np), Hai1 (Gb1WT), 3798 (Gh1np), and Huazhong 94-3130 (Gh1WT). The pre-squaring stage with five and six leaves for Xinhai16 (Gb2np), seven and eight leaves for Hai1 (Gb2WT), five leaves for 3798 (Gh2np), and six and seven leaves for Huazhong94-3130 (Gh2WT), respectively, all without any visible triangular floral bud, just in the forming stage of floral bud (flowering) and fruiting branch in the pre-squaring stage. The squaring stage with nine leaves for Xinhai16 (Gb3np), nine and ten leaves for Hai1 (Gb3WT), and eight to ten leaves for 3798 (Gh3np) and Huazhong94-3130 (Gh3WT), respectively, all with visible triangular floral buds in the field. These leaf numbers above refer to visual, expanded true leaves in cotton field^69^. The nodes of first fruiting branch we investigated for each line including Xinhai16, Hai1, 3798 and Huazhong 94-3130 were 6-7,8-9,5-6,7-8 true expanded leaves, respectively, in virtue of the different lines (varieties). The same RNA samples were used for sRNA-seq library construction, and we labelled the libraries with the prefix ‘mi’ as in ‘miGb1np’.

### Sample preparation and sequencing library construction

The mRNA and small RNA libraries were constructed as suggested by Illumina and were sequenced on a HiSeq 2000 platform. Briefly, total RNA was isolated using a Plant RNA EASYspin Plus Kit (Aidlab, Peking, China) according to the manufacturer’s instructions. Then, the total RNA of each sample was sent to BGI (Shenzhen, China) for library construction and sequencing. Finally, an Agilent 2100 Bioanalyzer and ABI Step One Plus Real-Time PCR System was used for the quantification and qualification of the sample library, after which the library was sequenced using an Illumina HiSeq™ 2000 platform.

### Alignment of RNA-seq reads to the reference genome

Reference genome sequences and annotations were downloaded from the Institute of cotton Research of CAAS (http://cgp.genomics.org.cn)^33^. Reads were aligned to the genome with no more than five mismatches using the SOAP aligner/SOAP2 software after low-quality reads (reads with unknown sequences ‘N’), adaptor sequence fragments, and empty reads were removed^70^. After passing the QC process of the alignment, the results were used for further analysis.

### Differential gene expression analysis

RPKM was used to obtain the relative levels of expression^71^,^72^. Differential expression analysis was performed using R packages of DESeq for comparisons among samples with three biological replicates^71^,^73^. We used a false discovery rate of <0.001 and an absolute value of the log 2 ratio >1 as the threshold for judging the significance of the gene expression differences^74^. The DEGs were further analysed by GO-Term Finder and path_finder for GO and KEGG enrichment^75^. The calculated *p*-value was Bonferroni corrected, taking the corrected *p*-value < 0.05 as a threshold for GO annotation. After the GO annotation was obtained for each gene, WEGO software was used to classify the genes by function and to determine the distribution of gene functions in the species at the macro level^76^. The DEGs were annotated using the Mercator web tool^77^ and then loaded into Mapman software for function enrichment analysis^78^,^79^,^80^. The Venn diagrams in this study were prepared using the function Venn diagram in R based on the gene list for each tissue type.

### Small RNA classification

After trimming adaptor sequences at the 5′ and 3′ ends of sequenced reads low-quality reads (reads with unknown sequences ‘N’), adaptor sequence fragments, and empty reads were filtered out. Reads were aligned to the genome using the SOAP aligner/SOAP2 software with parameter settings for perfect matches. Then, sRNA-seq reads were compared to miRNA databases including repeat RNA, miRBase (version 21, http://www.mirbase.org/ftp.shtml), GenBank (ftp://ftp.ncbi.nlm.nih.gov/genbank/), Rfam (version 11, http://rfam.janelia.org/), and snoRNA (http://www.mir2disease.org/) using the BlastN program (version 2.2.16). Mireap software (version 0.1) was used for novel miRNA prediction.

### Differential expression analysis of small RNAs and target prediction

Differentially expressed miRNAs between samples were identified using DESeq software^73^. The false discovery rate of <0.001 and an absolute value of the log2 ratio > 1 were used as the threshold for determining the significance of miRNA expression differences. miRNA target gene prediction was performed using psRobot and TargetFinder^81^,^82^,^83^,^84^,^85^. miRNA function categorisation and pathway enrichment were based on the GO and KEGG databases.

### Data validation by qPCR

Total RNA was extracted as described for DEG library preparation and sequencing. Total RNA (2 μg) from each sample was reverse transcribed in a 20 μL reaction using Reverse Transcriptase M-MLV (Takara). The sequences of the primers used are shown in Supplemental Table S8. The actin gene and/or UBQ7 gene of cotton (GeneBank accession No. is AY305733 and DQ116441) were used as internal control genes. qRT-PCR was performed using a SYBR Premix Ex Taq™ Kit (Takara) according to the manufacturer’s protocol. The 20 μL reaction volume consisted of forward and reverse primers (1 μL) SYBR premix Ex Taq II (10 μL), ddH_2_O (2.6 μL), cDNA (5 μL) and ROX Dye II (0.4 μL). The selected genes were verified using an ABI 7500 real-time PCR detection system with a cycling temperature of 60 °C and a single peak on the melting curve to ensure a single product. Relative transcript levels for each sample were obtained using the 2^−△△Ct^ method. At least three replicates were tested per sample.

## Additional Information

**Accession Numbers:** All the RNA-seq and small RNA raw data and processed data used in this study have been deposited at NCBI Gene Expression Omnibus under accession number GSE69025.

**How to cite this article**: Sun, Q. *et al*. To Be a Flower or Fruiting Branch: Insights Revealed by mRNA and Small RNA Transcriptomes from Different Cotton Developmental Stages. *Sci. Rep.* **6**, 23212; doi: 10.1038/srep23212 (2016).

## Supplementary Material

Supplementary Information

Supplementary Information

## Figures and Tables

**Table 1:** Pathway annotation to KEGG of pre-squaring stage-specific DEG in *G.barbadense.*

	Pathway	Gene number(2243)	Pvalue	Qvalue	Pathway ID
1	Photosynthesis-antenna proteins	27 (1.2%)	3.36E-20	9.69E-18	ko00196
2	Metabolic pathways	768 (34.24%)	7.32E-11	1.05E-08	ko01100
3	DNA replication	49 (2.18%)	4.77E-10	4.58E-08	ko03030
4	Amino sugar and nucleotide sugar metabolism	77 (3.43%)	1.83E-08	1.32E-06	ko00520
5	Starch and sucrose metabolism	117 (5.22%)	4.13E-08	2.38E-06	ko00500
6	Biosynthesis of secondary metabolites	413 (18.41%)	2.61E-07	1.25E-05	ko01110
7	Plant hormone signal transduction	224 (9.99%)	1.56E-06	6.40E-05	ko04075
8	Plant-pathogen interaction	256 (11.41%)	5.72E-06	2.06E-04	ko04626
9	Fructose and mannose metabolism	119 (5.31%)	7.83E-05	2.51E-03	ko00051
10	Alanine, aspartate and glutamate metabolism	43 (1.92%)	0.000155339	4.47E-03	ko00250
11	Tyrosine metabolism	110 (4.9%)	0.000375324	9.83E-03	ko00350
12	Cyanoamino acid metabolism	32 (1.43%)	0.000818251	1.96E-02	ko00460
13	Other glycan degradation	35 (1.56%)	0.001132179	2.19E-02	ko00511
14	Apoptosis	81 (3.61%)	0.001144075	2.19E-02	ko04210
15	Chloroalkane and chloroalkene degradation	25 (1.11%)	0.001226188	2.19E-02	ko00625
16	Cysteine and methionine metabolism	35 (1.56%)	0.001249362	2.19E-02	ko00270
17	Mismatch repair	29 (1.29%)	0.001290759	2.19E-02	ko03430
18	Carotenoid biosynthesis	35 (1.56%)	0.001746863	2.79E-02	ko00906
19	Pentose phosphate pathway	35 (1.56%)	0.002632152	3.08E-02	ko00030
20	NF-kappa B signaling pathway	78 (3.48%)	0.002674192	3.08E-02	ko04064
21	Drug metabolism-cytochrome P450	22 (0.98%)	0.002785508	3.08E-02	ko00982
22	Chlorocyclohexane and chlorobenzene degradation	8 (0.36%)	0.002830841	3.08E-02	ko00361
23	Toll-like receptor signaling pathway	82 (3.66%)	0.002855844	3.08E-02	ko04620
24	Atrazine degradation	5 (0.22%)	0.002867008	3.08E-02	ko00791
25	Neurotrophin signaling pathway	89 (3.97%)	0.002874946	3.08E-02	ko04722
26	Lipopolysaccharide biosynthesis	11 (0.49%)	0.002949529	3.08E-02	ko00540
27	Microbial metabolism in diverse environments	225 (10.03%)	0.003075122	3.08E-02	ko01120
28	Pertussis	84 (3.74%)	0.00308791	3.08E-02	ko05133
29	Glutathione metabolism	33 (1.47%)	0.003273129	3.08E-02	ko00480
30	Neuroactive ligand-receptor interaction	2 (0.09%)	0.003312447	3.08E-02	ko04080
31	Leishmaniasis	81 (3.61%)	0.003312899	3.08E-02	ko05140
32	Pyrimidine metabolism	166 (7.4%)	0.004005977	3.61E-02	ko00240
33	Photosynthesis	29 (1.29%)	0.004622528	4.03E-02	ko00195
34	Chagas disease (American trypanosomiasis)	83 (3.7%)	0.005105472	4.32E-02	ko05142
35	Nitrogen metabolism	26 (1.16%)	0.005286608	4.35E-02	ko00910
36	D-Alanine metabolism	3 (0.13%)	0.005595	4.38E-02	ko00473
37	Benzoate degradation	87 (3.88%)	0.005774368	4.38E-02	ko00362
38	Insulin signaling pathway	46 (2.05%)	0.005838026	4.38E-02	ko04910
39	Ascorbate and aldarate metabolism	30 (1.34%)	0.005933246	4.38E-02	ko00053
40	Metabolism of xenobiotics by cytochrome P450	21 (0.94%)	0.00619936	4.46E-02	ko00980
41	Measles	82 (3.66%)	0.006586086	4.58E-02	ko05162
42	Tuberculosis	98 (4.37%)	0.006679158	4.58E-02	ko05152

**Table 2:** Pathway annotation to KEGG of pre-squaring stage-specific DEG in *G. hirsutum.*

	Pathway	Gene number (40)	Pvalue	Qvalue	Pathway ID
1	Photosynthesis-antenna proteins	2 (5%)	0.001319016	0.09855362	ko00196
2	Isoquinoline alkaloid biosynthesis	2 (5%)	0.00323993	0.09855362	ko00950
3	Biosynthesis of secondary metabolites	13 (32.5%)	0.003478363	0.09855362	ko01110
4	Benzoxazinoid biosynthesis	2 (5%)	0.00914341	0.19429746	ko00402
5	Sesquiterpenoid and triterpenoid biosynthesis	2 (5%)	0.01309328	0.22258576	ko00909
6	Glycolysis/Gluconeogenesis	3 (7.5%)	0.01931683	0.27001173	ko00010
7	Chloroalkane and chloroalkene degradation	2 (5%)	0.02223626	0.27001173	ko00625
8	Fatty acid metabolism	2 (5%)	0.03182014	0.31785701	ko00071
9	Pentose and glucuronate interconversions	3 (7.5%)	0.03903229	0.31785701	ko00040
10	Retinol metabolism	1 (2.5%)	0.04813431	0.31785701	ko00830
11	Cell cycle-yeast	3 (7.5%)	0.04846708	0.31785701	ko04111
12	Glycerolipid metabolism	2 (5%)	0.04915537	0.31785701	ko00561
13	Two-component system	2 (5%)	0.04991741	0.31785701	ko02020
14	Tyrosine metabolism	4 (10%)	0.05235292	0.31785701	ko00350
15	Diterpenoid biosynthesis	2 (5%)	0.0561666	0.3182774	ko00904

**Table 3:** The Mapman function annotation of DEG at square initiating stage both in *G. hirsutum* and *G.barbadense.*

Gene id	BinName	Description (homology gene in Arabidopsis)
CotAD_58304	PS.lightreaction.other electron carrier (ox/red).ferredoxin	(at1g02180) ferredoxin-related
CotAD_35943	major CHO metabolism.degradation.starch.starch cleavage.beta amylase	(at4g15210) ATBETA-AMY
CotAD_16567	minor CHO metabolism.trehalose.potential TPS/TPP	(at4g17770) TPS5
CotAD_00103	glycolysis.cytosolic branch.non-phosphorylating glyceraldehyde 3-phosphate dehydrogenase (NPGAP-DH)	(at2g24270)aldehyde dehydrogenase 11A3 (ALDH11A3)
CotAD_10000	fermentation.ADH	(at1g77120) alcohol dehydrogenase 1 (ADH1)
CotAD_07955	TCA/org transformation.carbonic anhydrases	(at3g01500) carbonic anhydrase 1 (CA1)
CotAD_48130	mitochondrial electron transport/ATP synthesis.alternative oxidase	(at5g64210)alternative oxidase 2 (AOX2)
CotAD_71865	cell wall.modification	(at4g38210) expansin A20 (EXPA20)
CotAD_72788	cell wall.modification	(at4g17030) expansin-like B1 (EXLB1)
CotAD_27981	cell wall.modification	(at3g29030) expansin A5 (EXPA5)
CotAD_16307	cell wall.pectin*esterases.PME	(at1g02810) Plant invertase/pectin methylesterase inhibitor superfamily
CotAD_12986	metal handling	(at5g27690) Heavy metal transport/detoxification superfamily protein
CotAD_07016	secondary metabolism.isoprenoids.non-mevalonate pathway.geranylgeranyl pyrophosphate synthase	(at4g38460) geranylgeranyl reductase (GGR)
CotAD_24175	secondary metabolism.phenylpropanoids.lignin biosynthesis.F5H	(at5g07990) TRANSPARENT TESTA 7 (TT7)
CotAD_56508	secondary metabolism.sulfur-containing.glucosinolates	(at4g03070) AOP1
CotAD_44293	secondary metabolism.sulfur-containing.glucosinolates.synthesis.aliphatic.sulfotransferase	(at1g74090) desulfo-glucosinolate sulfotransferase 18 (SOT18)
CotAD_44293	secondary metabolism.sulfur-containing.glucosinolates.synthesis.indole.indole-3-methyl-desulfoglucosinolate sulfotransferase	(at1g74090) desulfo-glucosinolate sulfotransferase 18 (SOT18)
CotAD_15307	secondary metabolism.flavonoids.dihydroflavonols	(at5g24530) DOWNY MILDEW RESISTANT 6 (DMR6)
CotAD_24175	secondary metabolism.flavonoids.dihydroflavonols.flavonoid 3”-monooxygenase	(at5g07990)TRANSPARENT TESTA 7 (TT7)
CotAD_49345	hormone metabolism.abscisic acid.induced-regulated-responsive-activated	(at5g59220) highly ABA-induced PP2C gene 1 (HAI1)
CotAD_56508	hormone metabolism.gibberelin.synthesis-degradation	(at4g03070) AOP1
CotAD_12787	tetrapyrrole synthesis.unspecified	(at1g17100) SOUL heme-binding family protein
CotAD_40314	stress.biotic	(at3g12500) basic chitinase (HCHIB)
CotAD_73916	stress.biotic.PR-proteins	(at1g61180) LRR and NB-ARC domains-containing disease resistance protein
CotAD_52530	stress.biotic.PR-proteins.proteinase inhibitors.trypsin inhibitor	(at1g17860) Kunitz family trypsin and protease inhibitor protein
CotAD_22468	stress.biotic.PR-proteins.proteinase inhibitors.trypsin inhibitor	(at1g17860) Kunitz family trypsin and protease inhibitor protein
CotAD_41277	stress.abiotic.drought/salt	(at1g30360) early-responsive to dehydration 4 (ERD4)
CotAD_68015	stress.abiotic.unspecified	(at5g66590) CAP superfamily protein
CotAD_24175	misc.cytochrome P450	(at5g07990)TRANSPARENT TESTA 7 (TT7)
CotAD_65900	misc.short chain dehydrogenase/reductase (SDR)	(at4g11410) NAD(P)-binding Rossmann-fold superfamily protein
CotAD_44293	misc.sulfotransferase	(at1g74090)desulfo-glucosinolate sulfotransferase 18 (SOT18)
CotAD_09729	misc.GDSL-motif lipase	(at5g40990) GDSL lipase 1 (GLIP1)
CotAD_26978	misc.GDSL-motif lipase	(at5g40990) GDSL lipase 1 (GLIP1)
CotAD_22031	RNA.regulation of transcription.AP2/EREBP, APETALA2/Ethylene-responsive element binding protein family	(at1g13260) related to ABI3/VP1 1 (RAV1)
CotAD_21647	RNA.regulation of transcription.CCAAT box binding factor family, HAP5	(at5g43250) “nuclear factor Y, subunit C13” (NF-YC13)
CotAD_47245	RNA.regulation of transcription.MADS box transcription factor family	(at2g22540) SHORT VEGETATIVE PHASE (SVP)
CotAD_32839	RNA.regulation of transcription.MYB-related transcription factor family	(at1g01060) LATE ELONGATED HYPOCOTYL (LHY)
CotAD_30985	RNA.regulation of transcription.MYB-related transcription factor family	(at1g18330) EARLY-PHYTOCHROME-RESPONSIVE1 (EPR1)
CotAD_02759	RNA.regulation of transcription.TCP transcription factor family	(at3g18550) BRANCHED 1 (BRC1)
CotAD_00235	RNA.regulation of transcription.Aux/IAA family	(at1g51950) indole-3-acetic acid inducible 18 (IAA18)
CotAD_14148	RNA.regulation of transcription.Psudo ARR transcription factor family	(at5g24470) pseudo-response regulator 5 (PRR5)
CotAD_54816	RNA.regulation of transcription.putative transcription regulator	(at1g04020)breast cancer associated RING 1 (BARD1)
CotAD_04662	RNA.regulation of transcription.putative transcription regulator	(at2g29570)proliferating cell nuclear antigen 2 (PCNA2)
CotAD_55265	RNA.regulation of transcription.SET-domain transcriptional regulator family	(at5g24330)ARABIDOPSIS TRITHORAX-RELATED PROTEIN 6 (ATXR6)
CotAD_00066	RNA.regulation of transcription.unclassified	(at5g10770) Eukaryotic aspartyl protease family protein
CotAD_65745	DNA.synthesis/chromatin structure	(at1g26840) origin recognition complex protein 6 (ORC6)
CotAD_49345	protein.postranslational modification	(at5g59220) highly ABA-induced PP2C gene 1 (HAI1)
CotAD_00063	protein.degradation.aspartate protease	(at5g10770) Eukaryotic aspartyl protease family protein
CotAD_64010	protein.degradation.ubiquitin.E3.RING	(at4g01270) RING/U-box superfamily protein
CotAD_72041	signalling.receptor kinases.leucine rich repeat VIII.VIII-2	(at1g56130) Leucine-rich repeat transmembrane protein kinase
CotAD_58997	signalling.phosphinositides.phosphatidylinositol-4-phosphate 5-kinase	(at1g71010) FORMS APLOID AND BINUCLEATE CELLS 1C (FAB1C)
CotAD_25160	signalling.MAP kinases	(at3g51630) with no lysine (K) kinase 5 (WNK5)
CotAD_06239	development.unspecified	(at3g59550)SYN3
CotAD_07322	development.unspecified	(at3g59550) SYN3
CotAD_42150	development.unspecified	(at4g19450) Major facilitator superfamily protein
CotAD_50063	development.unspecified	(at1g69490) NAC-like, activated by AP3/PI (NAP)
CotAD_07846	transport.amino acids	(at5g40780) LHT1 (lysine histidine transporter)
CotAD_40809	transport.unspecified cations	(at5g64560)magnesium transporter 9 (MGT9)
CotAD_61138	transport.misc	(at5g52450) MATE efflux family protein
CotAD_54187	not assigned.no ontology.hydroxyproline rich proteins	(at4g01050)thylakoid rhodanese-like (TROL)
CotAD_43563	not assigned.unknown	no original description
CotAD_18636	not assigned.unknown	(at5g40460) unknown protein
CotAD_17275	not assigned.unknown	(at3g48490) unknown protein
CotAD_29961	not assigned.unknown	(at5g05250) unknown protein
CotAD_23730	not assigned.unknown	(at5g56850) unknown protein
CotAD_26649	not assigned.unknown	(at2g38640)unknown function (DUF567)
CotAD_34939	not assigned.unknown	(at3g01680)unknown protein
CotAD_46956	not assigned.unknown	(gnl|cdd|36433) no description available
CotAD_56138	not assigned.unknown	(at3g56870) unknown protein
CotAD_48331	not assigned.unknown	(gnl|cdd|36433) no description available
CotAD_20876	not assigned.unknown	(at4g28310) unknown protein
CotAD_22049	not assigned.unknown	(at3g18440) aluminum-activated malate transporter 9 (ALMT9)
CotAD_74231	not assigned.unknown	(at4g28310) unknown protein
CotAD_40867	not assigned.unknown	(at5g21940) unknown protein

**Table 4:** Identified targets for known miRNAs in cotton.

	miR-name	log2Ratio	TargetID	log2Ratio	type	Target annotation
Gb1np/Gb1WT	miR9484	18.30964	CotAD_21580	−1.13888	up-down	unkown
			CotAD_34848	−2.5911	up-down	LRR family protein
	miR9674a-3p	−19.9987	CotAD_56347	1.333495	down-up	EMBRYO DEFECTIVE 976 (EMB976)
Gb2np/Gb2WT	miR5291a	−16.6434	CotAD_63772	1.518275	down-up	Glycosyl hydrolase family protein
	miR5537	19.1318	CotAD_07870	−1.05164	up-down	chloride channel A (CLC-A)
			CotAD_32730	−1.01925	up-down	Tetratricopeptide repeat (TPR)-like superfamily protein
			CotAD_72047	−1.34026	up-down	HXXXD-type acyl-transferase family protein
			CotAD_73186	−2.15217	up-down	HXXXD-type acyl-transferase family protein
	miR5641	−18.4987	CotAD_30697	3.752667	down-up	glycosyl hydrolase 9B18 (GH9B18)
			CotAD_35367	2.500502	down-up	SEPALLATA3 (SEP3)
	miR4393b	−19.5048	CotAD_41814	1.089024	down-up	cellulose-synthase like D2 (CSLD2)
	miR1077-5p	22.57754	CotAD_07688	−1.3105	up-down	unkown
			CotAD_35213	−1.28959	up-down	unkown
			CotAD_47342	−1.17882	up-down	unkown
			CotAD_48531	−1.29591	up-down	RRNA intron-encoded homing endonuclease
			CotAD_53140	−1.34414	up-down	unkown
			CotAD_70473	−1.13899	up-down	unkown
	miR156i-3p	17.38975	CotAD_31099	−2.04149	up-down	unkown
			CotAD_54581	−2.46349	up-down	galacturonosyltransferase 12 (GAUT12)
	miR166i	21.68899	CotAD_39504	−2.67928	up-down	unkown
			CotAD_75643	−1.8728	up-down	homeobox gene 8 (HB-8)
	miR9484	21.06244	CotAD_01114	−1.16244	up-down	NB-ARC domain-containing disease resistance protein
			CotAD_16057	−1.32682	up-down	unkown
			CotAD_21416	−1.28186	up-down	Late embryogenesis abundant protein, LEA-5
			CotAD_25778	−1.15489	up-down	pleiotropic drug resistance 5 (PDR5)
			CotAD_26434	−1.74762	up-down	nitrate transporter 1:2 (NRT1:2)
			CotAD_34848	−2.29922	up-down	LRR family protein
			CotAD_40632	−1.10811	up-down	allantoate amidohydrolase (AAH)
			CotAD_44160	−1.96883	up-down	receptor like protein 35 (RLP35)
			CotAD_47999	−1.29278	up-down	pleiotropic drug resistance 5 (PDR5)
			CotAD_68929	−2.75838	up-down	unkown
	miR862-3p	−16.5445	CotAD_40927	1.124491	down-up	unkown
	miR482d	17.53683	CotAD_34619	−5.55857	up-down	RECOGNITION OF PERONOSPORA PARASITICA 8 (RPP8)
	miR5298d	−16.4595	CotAD_45973	2.497785	down-up	unkown
			CotAD_67595	4.281629	down-up	Cysteine/Histidine-rich C1 domain family protein
Gb3np/Gb3WT	miR7502	−4.33643	CotAD_35035	1.416654	down-up	FRAGILE FIBER 1 (FRA1)
	miR482b	19.36951	CotAD_59175	−1.5279	up-down	NB-ARC domain-containing disease resistance protein
	miR167a-5p	25.12849	CotAD_21735	−1.82028	up-down	GDSL-like Lipase/Acylhydrolase superfamily protein
			CotAD_24173	−2.20715	up-down	Flavonoid 3′-monooxygenase
	miR829-5p	−17.6664	CotAD_20201	3.869985	down-up	Disease resistance protein (TIR-NBS-LRR class) family
	miR5641	−18.9006	CotAD_30080	1.385631	down-up	SABRE (SAB)
			CotAD_37128	1.089237	down-up	unkown
			CotAD_38231	1.789472	down-up	PARVUS (PARVUS)
			CotAD_65515	1.039363	down-up	P-loop containing nucleoside triphosphate hydrolases superfamily protein
	miR1159.1	21.12174	CotAD_28336	−14.1677	up-down	unkown
	miR157a-5p	−3.41905	CotAD_19197	5.586874	down-up	O-methyltransferase family protein
Gh1np/Gh1WT	miR9484	−18.1864	CotAD_52129	1.875941	down-up	probable E3 ubiquitin-protein ligase LOG2-like
Gh3np/Gh3WT	miR5537	18.61224	CotAD_28668	−3.57269	up-down	wall associated kinase-like 1 (WAKL1)
	miR6103-3p	−20.0962	CotAD_05723	1.008805	down-up	cysteine-rich RLK (RECEPTOR-like protein kinase) 25 (CRK25)
			CotAD_15382	1.28365	down-up	guanylyl cyclase 1 (GC1)
			CotAD_57423	3.435504	down-up	cysteine-rich RLK (RECEPTOR-like protein kinase) 26 (CRK26)
			CotAD_57424	1.427739	down-up	cysteine-rich RLK (RECEPTOR-like protein kinase) 10 (CRK10)
			CotAD_75962	1.142944	down-up	phragmoplast orienting kinesin 2 (POK2)
	miR2950	−3.81938	CotAD_23640	1.161408	down-up	hydroxyproline-rich glycoprotein family protei
	miR5021	−17.16	CotAD_02433	2.113803	down-up	MYB
			CotAD_05722	1.87195	down-up	cysteine-rich RLK (RECEPTOR-like protein kinase) 25 (CRK25)
			CotAD_08430	1.194253	down-up	Vacuolar import/degradation, Vid27-related protein
			CotAD_08496	1.074783	down-up	DREB1C
			CotAD_13093	1.070816	down-up	lectin protein kinase family protein
			CotAD_13340	1.687647	down-up	AAA-ATPase 1 (AATP1)
			CotAD_19878	1.284197	down-up	senescence-related gene 3 (SRG3)
			CotAD_28123	1.288063	down-up	early nodulin-like protein 1 (ENODL1)
			CotAD_31169	2.538615	down-up	sucrose-proton symporter 2 (SUC2)
			CotAD_35030	1.129006	down-up	WRKY transcription factor 26
			CotAD_38978	2.753387	down-up	dehydrin 2
			CotAD_68811	2.057678	down-up	plastid developmental protein DAG
			CotAD_72901	1.577081	down-up	FERONIA (FER)
	miR156a-5p	−20.6349	CotAD_28172	1.611284	down-up	PAS domain-containing protein tyrosine kinase family protein
	miR414	−18.2483	CotAD_05693	2.220604	down-up	unkown
			CotAD_08496	1.074783	down-up	DREB1C
			CotAD_20312	1.280126	down-up	plant intracellular ras group-related LRR 2
			CotAD_35408	1.248969	down-up	global transcription factor group E4 (GTE4)
			CotAD_39897	4.054389	down-up	ABA REPRESSOR1 (ABR1)
			CotAD_40639	2.371314	down-up	Calcium-binding EF-hand family protein
			CotAD_41567	1.228057	down-up	NBS-LRR resistance protein RGH2
			CotAD_42550	1.495769	down-up	unkown
			CotAD_53959	3.793863	down-up	CYCLIN D1;1 (CYCD1;1)
			CotAD_57315	4.286361	down-up	unkown
			CotAD_62266	2.179486	down-up	receptor like protein 13 (RLP13)
			CotAD_63349	4.788476	down-up	formin homology5 (Fh5)
