# Supplementary material for: To Be a Flower or Fruiting Branch: Insights Revealed by mRNA and Small RNA Transcriptomes from Different Cotton Developmental Stages
Source: Sci Rep. 2016 Mar 17;6:23212. doi: 10.1038/srep23212 (PMC4794708; doi:10.1038/srep23212)
Supplement: Supplementary Information [file srep23212-s1.pdf]

Supplementary Information

Title:

To Be a Flower or Fruiting Branch: Insights Revealed by mRNA and Small RNA Transcriptomes from Different Cotton Developmental Stages

Author:

Quan Sun[1,2*], Xiongming Du[3*], Chaowei Cai[1], Lu Long[1], Sai Zhang[1], Peng Qiao[1], Weina Wang[2], Kexue Zhou[1], Guanghao Wang[1], Xin Liu[1], Hui Zhang[1], Shuaipeng Geng[1], Can Yang[2], Wei Gao[1], Jianchuan Mo[1], Chen Miao[1], Chunpeng Song[1**], Yingfan Cai[1**]

** Correspondence: cyf@henu.edu.cn, songcp@henu.edu.cn

Supplemental Figure S1

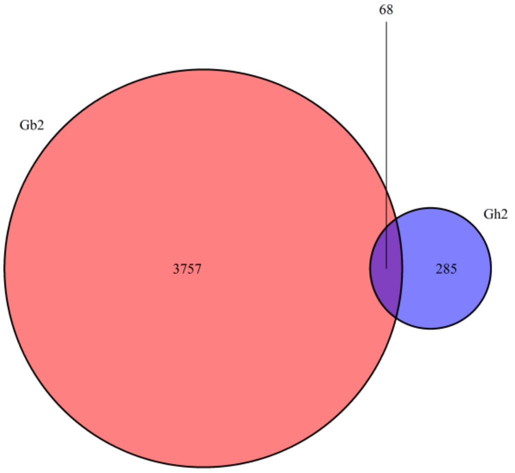

Supplemental Figure S1|Venn diagram analyses of pre-squaring stage specific expression genes in *G.barbadense* (Gb2) and *G. hirsutum* (Gh2).

Supplemental Figure S2

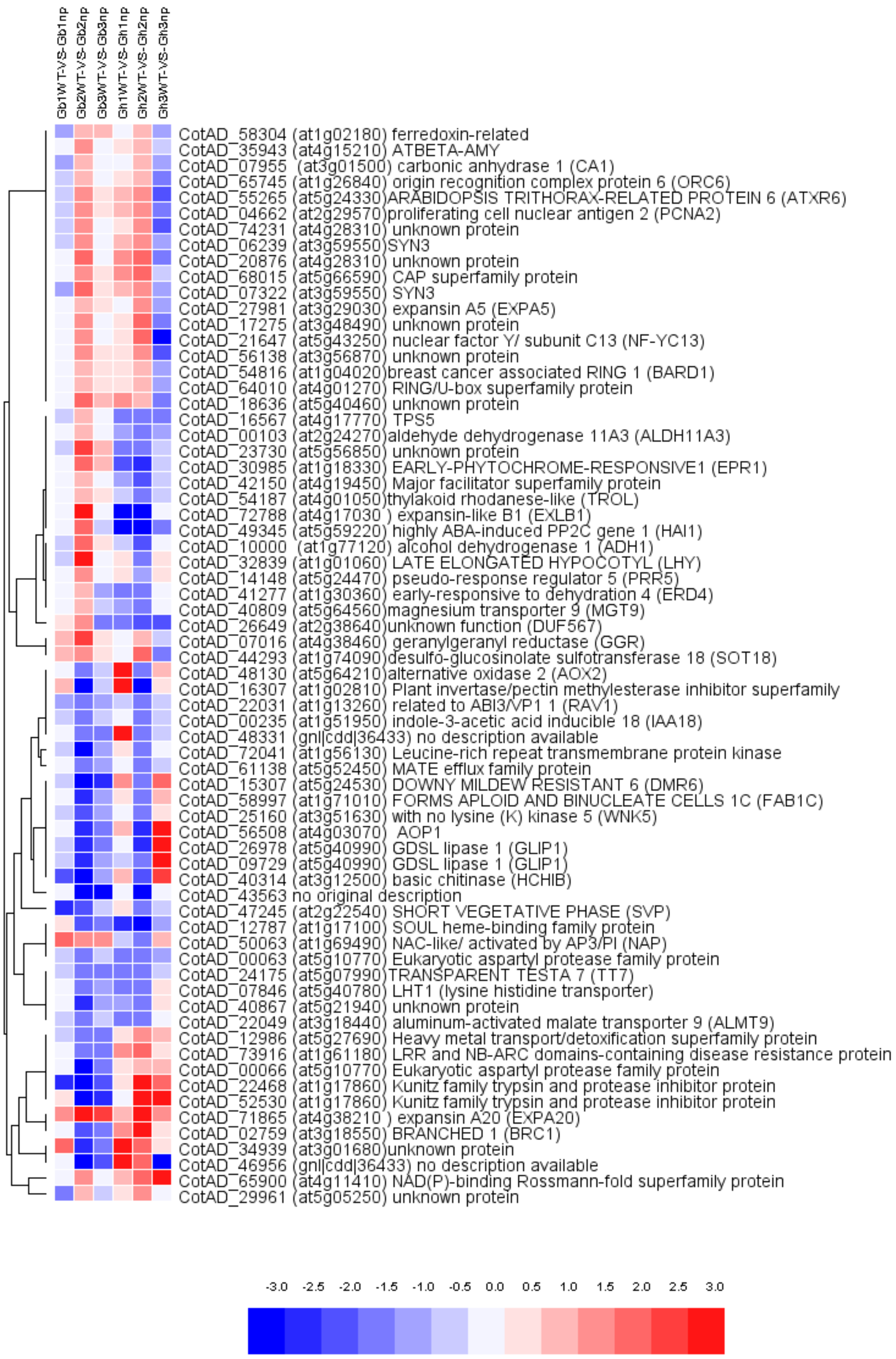

Supplemental Figure S2 | Expression profiles for stage-specific DEGs in square initiating stage both in *G. hirsutum* and *G.barbadense*. The bar show log2 value of fold change.

Supplemental Figure S3

A B

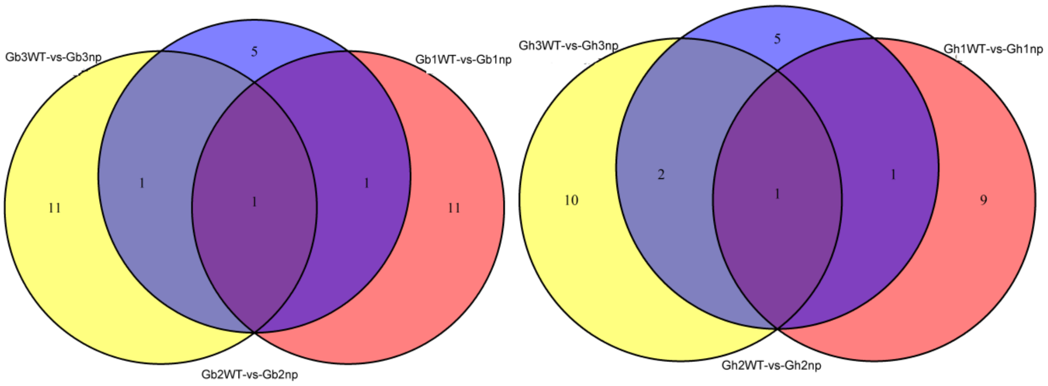

Supplemental Figure S3 | Venn diagram analyses of stage-specific expression novel miRNAs in *G.barbadense* (A) *G. hirsutum* (B).

Supplemental Figure S4

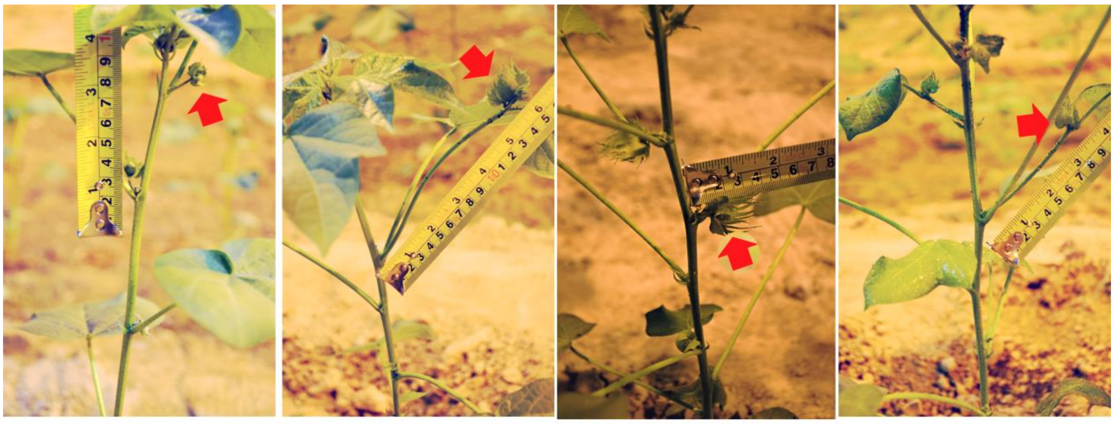

Xinhai16 | Hai1 | 3798 | Huazhong 94-3130

Supplemental Figure S4 | The buds grown in different positions of the plant for two different branch types of cotton, nulliplex-branch mutant: 3798 and Xinhai 16, wild type: Huazhong94-3130 and Hai1

Supplemental Figure 5

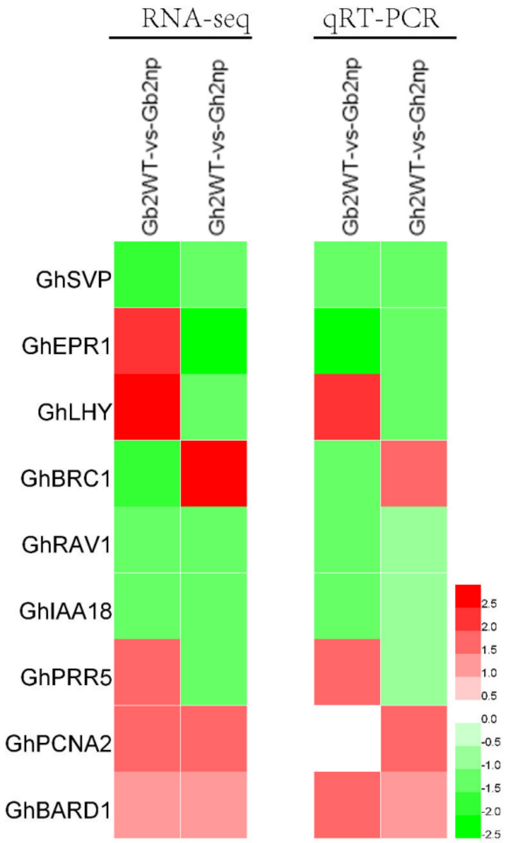

Supplemental Figure S5 | Confirmation of RNA-seq expression profiles with qPCR. Left showed expression ratios, as log2 (fold change) for RNA-seq in six compared groups. Right showed the expression ratios, also as log2 (fold change) for qRT-PCR in six compared groups.

Supplemental Table S1 | Raw data statistic of RNA-seq

Samples	Total Raw Reads	Total Clean Reads	Total Clean Nucleotides (nt)	Q20 percentage	N percentage	GC percentage
Gb1WT-1	55,582,284	52,864,154	4,757,773,860	98.33%	0.01%	44.23%
Gb1WT-2	54,681,700	51,962,266	4,676,603,940	98.28%	0.01%	44.31%
Gb1WT-3	55,823,910	53,033,216	4,772,989,440	98.32%	0.01%	44.41%
Gb1np-1	58,571,526	53,135,140	4,782,162,600	98.18%	0.01%	44.24%
Gb1np-2	57,387,014	54,464,656	4,901,819,040	98.32%	0.01%	44.37%
Gb1np-3	54,024,896	51,414,774	4,627,329,660	98.32%	0.01%	44.15%
Gb2WT-1	55,042,908	52,265,610	4,703,904,900	98.04%	0.01%	43.37%
Gb2WT-2	58,489,636	55,355,962	4,982,036,580	97.97%	0.01%	43.51%
Gb2WT-3	57,670,542	54,880,690	4,939,262,100	98.07%	0.01%	43.34%
Gb2np-1	57,907,284	54,917,224	4,942,550,160	97.62%	0.01%	43.29%
Gb2np-2	58,507,324	55,517,736	4,996,596,240	97.65%	0.01%	43.43%
Gb2np-3	56,863,582	53,880,600	4,849,254,000	97.61%	0.01%	43.37%
Gb3WT-1	57,576,230	54,971,746	4,947,457,140	98.14%	0.01%	43.89%
Gb3WT-2	57,580,500	54,582,626	4,912,436,340	97.58%	0.01%	43.58%
Gb3WT-3	54,580,784	51,652,392	4,648,715,280	97.56%	0.01%	43.59%
Gb3np-1	56,264,006	53,801,290	4,842,116,100	98.21%	0.01%	43.60%
Gb3np-2	53,761,796	51,352,526	4,621,727,340	98.15%	0.01%	43.61%
Gb3np-3	54,397,962	51,872,336	4,668,510,240	98.15%	0.01%	43.51%
Gh1WT-1	54,451,984	51,838,904	4,665,501,360	98.34%	0.01%	44.04%
Gh1WT-2	56,360,552	53,534,544	4,818,108,960	98.29%	0.01%	44.46%
Gh1WT-3	54,794,486	51,533,632	4,638,026,880	98.28%	0.01%	44.30%
Gh1np-1	54,119,498	51,174,464	4,605,701,760	97.99%	0.01%	45.11%
Gh1np-2	54,225,148	51,484,394	4,633,595,460	98.06%	0.01%	44.19%
Gh1np-3	58,869,832	55,545,094	4,999,058,460	97.97%	0.01%	44.47%
Gh2WT-1	54,118,698	51,215,100	4,609,359,000	97.63%	0.01%	43.38%
Gh2WT-2	57,748,844	54,860,278	4,937,425,020	98.03%	0.01%	43.55%
Gh2WT-3	58,154,564	55,324,496	4,979,204,640	98.03%	0.01%	43.49%
Gh2np-1	57,105,044	54,029,086	4,862,617,740	97.56%	0.01%	43.39%
Gh2np-2	59,847,754	54,979,614	4,948,165,260	97.54%	0.01%	43.55%
Gh2np-3	53,905,896	51,163,388	4,604,704,920	97.65%	0.01%	43.41%
Gh3WT-1	55,026,558	52,429,324	4,718,639,160	98.08%	0.01%	43.86%
Gh3WT-2	55,881,492	53,145,738	4,783,116,420	98.07%	0.01%	43.57%
Gh3WT-3	53,666,326	51,267,494	4,614,074,460	98.18%	0.01%	43.94%
Gh3np-1	57,848,924	54,620,742	4,915,866,780	97.95%	0.01%	43.46%
Gh3np-2	55,340,148	52,414,004	4,717,260,360	97.94%	0.01%	43.45%
Gh3np-3	56,159,378	53,293,290	4,796,396,100	97.99%	0.01%	43.39%

Supplemental Table S2 | GO annotation of pre-squaring stage-specific DEG in *G.barbadense*

	Gene Ontology term	Gene number	P-value
CC(1510)	chloroplast	128(8.5%)	2.57E-16
	chloroplast part	124(8.2%)	8.80E-16
	thylakoid	125(8.3%)	1.05E-15
	chloroplast thylakoid	111(7.4%)	5.75E-15
	plastid thylakoid	113(7.5%)	4.84E-14
	organelle subcompartment	113(7.5%)	6.15E-14
	photosynthetic membrane	46(3.0%)	2.32E-11
	photosystem	39(2.6%)	1.75E-10
	thylakoid part	50(3.3%)	1.22E-09
	chloroplast stroma	27(1.8%)	4.62E-09
	plastid part	200(13.2%)	9.52E-09
	plastid	377(25.0%)	1.02E-06
	plastid stroma	90(6.0%)	0.00058
	photosystem I	9(0.6%)	0.00309
	intrinsic to membrane	229(15.2%)	0.00321
	thylakoid light-harvesting complex	4(0.3%)	0.00567
	external encapsulating structure	96(6.4%)	0.01696
	nucleotide-excision repair complex	3(0.2%)	0.02131
	plastid envelope	81(5.4%)	0.02328
	germ cell nucleus	4(0.3%)	0.03625
	anchored to membrane	20(1.3%)	0.04227
MF(2024)	tetrapyrrole binding	26(1.3%)	2.36E-07
	oxidoreductase activity, acting on CH or CH2 groups, disulfide as acceptor	8(0.4%)	2.36E-06
	hydrolase activity, acting on glycosyl bonds	104(5.1%)	4.38E-05
	oxidoreductase activity, acting on CH or CH2 groups	8(0.4%)	0.00243
	cofactor binding	90(4.4%)	0.00431
	oxidoreductase activity, acting on single donors with incorporation of molecular oxygen	30(1.5%)	0.00437
	DNA-directed RNA polymerase activity	4(0.2%)	0.00455
	dioxygenase activity	36(1.8%)	0.0093
	carbon-carbon lyase activity	37(1.8%)	0.02418
	oxidoreductase activity	340(16.8%)	0.02479
	hydrolase activity, hydrolyzing O-glycosyl compounds	69(3.4%)	0.04321
	transmembrane transporter activity	165(8.2%)	0.0445
BP(1787)	nitrogen compound transport	65(3.6%)	8.76E-09
	generation of precursor metabolites and energy	86(4.8%)	4.69E-08
	amide transport	31(1.7%)	1.98E-07
	response to radiation	104(5.8%)	9.68E-07
	photosynthesis	30(1.7%)	1.84E-06
	photosynthesis, light reaction	28(1.6%)	2.71E-06
	response to light stimulus	80(4.5%)	2.79E-06
	response to light intensity	31(1.7%)	4.86E-06
	peptide transport	26(1.5%)	2.93E-05
	response to abiotic stimulus	183(10.2%)	5.93E-05
	photosynthetic electron transport chain	20(1.1%)	0.00012
	response to stimulus	565(31.6%)	0.00037
	anion transport	46(2.6%)	0.00049
	glucan metabolic process	47(2.6%)	0.00059
	polysaccharide metabolic process	60(3.4%)	0.00076
	carbohydrate metabolic process	122(6.8%)	0.00159
	response to red or far red light	35(2.0%)	0.00462
	response to oxygen-containing compound	72(4.0%)	0.00945
	response to far red light	5(0.3%)	0.03695
	cellular carbohydrate metabolic process	55(3.1%)	0.03749

Supplemental Table S3 | GO annotation of pre-squaring stage-specific DEG in *G. hirsutum*

	Gene Ontology term	Gene number	P-value
MF(176)	sesquiterpene synthase activity	5(2.8%)	0.00018
	carbon-oxygen lyase activity, acting on phosphates	6(3.4%)	0.00022
	terpene synthase activity	5(2.8%)	0.00096
	oxidoreductase activity	45(25.6%)	0.00274
	iron ion binding	15(8.5%)	0.01118
	ion binding	53(30.1%)	0.01873
	amylase activity	4(2.3%)	0.03057
	carbon-oxygen lyase activity	8(4.5%)	0.04376

Supplemental Table S4 | Statistical analysis of sequencing reads and distribution of small RNAs from twelve sRNA libraries in cotton

		Total	Matched	miRNA	rRNA	snRNA	snoRNA	tRNA	unann
**miGb1WT**	Uniqu(%)	4474033	3039685(67)	31528(0.72)	45558(1.17)	1392(0.03)	808(0.02)	5763(0.15)	4160090(90.25)
	Total(%)	12078303	9727581(80)	3655768(30.02)	579837(4.75)	2836(0.02)	1709(0.01)	135522(1.11)	6459510(53.1)
**miGb1np**	Uniqu(%)	4172688	2865411(68)	29108(0.66)	37979(0.98)	1347(0.04)	759(0.02)	4997(0.13)	3786021(90.59)
	Total(%)	11585879	9557316(82)	3921513(33.86)	401589(3.47)	2997(0.02)	1619(0.01)	99631(0.86)	5934357(51.19)
**miGb2WT**	Uniqu(%)	4699514	3203195(68)	25890(0.55)	46080(1.11)	1569(0.04)	899(0.02)	5783(0.14)	4284765(90.89)
	Total(%)	11418835	8813601(77)	987225(8.64)	810271(7.09)	3739(0.03)	2907(0.02)	163213(1.43)	7663450(67.12)
**miGb2np**	Uniqu(%)	4563616	3087176(67)	26342(0.6)	56194(1.53)	1603(0.04)	970(0.03)	7255(0.19)	4135888(89.97)
	Total(%)	11824530	9262478(78)	1103724(9.33)	1119070(9.43)	3701(0.03)	3576(0.03)	218339(1.84)	7335940(62.1)
**miGb3WT**	Uniqu(%)	5840522	3983736(68)	35448(0.61)	36298(0.56)	2082(0.03)	1154(0.02)	5830(0.09)	5332433(91.38)
	Total(%)	11548644	8770452(75)	976855(8.44)	386402(3.35)	4694(0.04)	2624(0.02)	103323(0.89)	8427033(72.97)
**miGb3np**	Uniqu(%)	4251084	2847955(66)	25030(0.62)	54580(1.36)	1822(0.05)	924(0.02)	7027(0.18)	3849375(90.33)
	Total(%)	11909011	9298965(78)	1276813(10.72)	1104241(9.27)	4954(0.04)	3387(0.03)	205191(1.72)	7202315(60.47)
**miGh1WT**	Uniqu(%)	5135991	4127043(80)	32802(0.59)	41372(0.78)	1341(0.02)	735(0.01)	4812(0.09)	4672749(91.29)
	Total(%)	11802342	10331167(87)	2397414(20.32)	418142(3.54)	2706(0.02)	1388(0.01)	101884(0.86)	7258829(61.48)
**miGh1np**	Uniqu(%)	5081157	4053379(79)	33310(0.66)	36964(0.75)	1318(0.03)	731(0.02)	4376(0.1)	4621375(90.87)
	Total(%)	12113952	10568966(87)	2599202(21.49)	357448(2.94)	2721(0.02)	1397(0.01)	104803(0.86)	7331767(60.50)
**miGh2WT**	Uniqu(%)	4693705	3774429(80)	25446(0.56)	50658(1.15)	1686(0.04)	868(0.02)	6190(0.14)	4243476(90.11)
	Total(%)	12016249	10370737(86)	1287205(10.71)	1003192(8.35)	3824(0.03)	2511(0.02)	188202(1.57)	7382912(61.42)
**miGh2np**	Uniqu(%)	4457930	3577845(80)	25424(0.55)	54198(1.04)	1651(0.03)	859(0.02)	6407(0.13)	4008255(90.06)
	Total(%)	11546843	10029483(86)	1200926(10.43)	978732(8.48)	3779(0.03)	2799(0.02)	188002(1.63)	7001306(60.65)
**miGh3WT**	Uniqu(%)	6349325	5054794(79)	35520(0.57)	31028(0.38)	1564(0.02)	770(0.01)	4017(0.05)	5804822(91.61)
	Total(%)	11505129	9748728(84)	765647(6.65)	240728(2.09)	3034(0.02)	1173(0.01)	45615(0.4)	8721796(75.79)
**miGh3np**	Uniqu(%)	4715587	3775532(80)	24854(0.5)	48733(0.85)	1799(0.03)	847(0.01)	6033(0.09)	4273328(91.28)
	Total(%)	11623897	10012854(86)	1010827(8.69)	788646(6.82)	4785(0.04)	2509(0.02)	170575(1.47)	7588006(65.16)

Supplemental Table S5 | The stage-specific DEM at pre-squaring stage in *G.barbadense*

miR_name	Sequence	log2Ratio(miGb2np/miGb2WT)	P-value	FDR
miR156c-3p	TGCTTACTTCTCTTCTGTCGGC	-17.3452	1.07E-05	0.000205
miR5298d	TGGGATGGTTGAAATGAAAAA	-16.4595	0.000405	0.005034
miR482d	TTTCCTATGCCCCCCATTCCAC	17.53683	4.36E-06	9.34E-05
miR2913	GTAGGTCGGGGATGCAAGTTGGAG	-17.8921	4.78E-07	1.15E-05
miR7496a	ATGACCAAATTGATAGAATGTGTA	-16.9319	7.69E-05	0.001287
miR5072	CGTTCCCCAGCGGAGTCGCCA	16.821	0.000119	0.001767
miR4354	CAACTCTGGATGGTCCAAAGCGGC	-16.8276	0.000112	0.001729
miR5537	TATGTTTTTAGGATCGTTGT	19.1318	5.00E-11	2.14E-09
miR4393b	TTGAAAAGGGATACAGATGACGC	-19.5048	2.37E-12	1.30E-10
miR5150-3p	AGCAGCTGCAGATCGTCAGAAGTC	-17.0137	5.98E-05	0.001046
miR5293	GATGAAGGTAGAAGGAACAGAAGA	4.970722	4.32E-05	0.000793
miR5291a	GTTGAGGATGGATTTGGATGGGTG	-16.6434	0.000211	0.003006
miR862-3p	ATATGTGAGATGCTGACTTGAAGA	-16.5445	0.000301	0.00399
miR1077-5p	TTGAAGTGTTCGGATCGCGGC	22.57754	1.03E-25	1.32E-23
miR156i-3p	TGCTCACTTCTCTTCTGTCAGC	17.38975	8.96E-06	0.000181
miR171c-5p	ATTGGTGCGGTTCAATCAGAT	17.68036	1.76E-06	3.98E-05

Supplemental Table S6 | The stage-specific DEM at pre-squaring stage in *G. hirsutum*

miR_name	sequence	log2Ratio(miGh2np/miGh2WT)	P-value	FDR
miR166c	TATATGATCTCGGACCAGGCT	-19.4183	6.60E-09	3.80E-07
miR1520f-3p	AACCAGACAGTGACACATGACATA	-16.5558	0.000382	0.006154
miR5210	TAGGAATGTTAGGATATTAAGGTT	-6.85011	1.91E-05	0.000454
miR4354	CAACTCTGGATGGTCCAAAGCGGC	-16.5785	0.000383	0.006154
miR2619b-5p	TATGTTTTGGATTATCTTATGGCA	-16.4968	0.000553	0.007965
miR5672	CAGGGTAGTGGAAGCAATTGCGA	-18.8299	1.04E-07	4.21E-06
miR398b-5p	TGAGTTGCCTATAGAACACACGGT	11.78364	8.90E-26	3.59E-23
miR832-5p	TACTGGTGGTCGGGAATGAAA	17.32932	2.54E-05	0.000569
miR7804-5p	AGTGAGTGTCTGATCGAATCGAAT	20.83867	4.59E-13	4.63E-11
miR5054	GTTTCCCACGGACGGCGCCA	20.69966	1.61E-12	1.30E-10
miR8613	ATCAAAGTTGGATGACTGTTGGTG	18.65141	1.64E-07	6.03E-06
miR1119	CGAGCACGGCGTGACTTGTCAG	-16.5858	0.000412	0.006154
miR8710a	ATACATCGAACTTTCTCCACA	17.4994	1.36E-05	0.000341
miR4249	TGATTTTGAGAAGTATGATGGGA	16.96606	8.45E-05	0.00148
miR6475	TCTTGAGACAAGCAAAGAACAC	-22.4831	2.87E-19	5.77E-17
miR2950-5p	TTCCATCTCTTGCACACTGGA	-17.7163	7.68E-06	0.000238
miR1510a-3p	CGGTAGGATCAGGTAAATCAAC	-16.5906	0.000406	0.006154
miR5137	AGCGGAGAAGATCGTTTGGT	17.25097	3.50E-05	0.000742

Supplement Table S7 | The list of DEGs enriched in two KEGG pathways. The up- or down-regulation information of the DEGs were showed in supplements data set 2 and 5.

	The enriched DEGs Gene ID in Gb2WT-vs-Gb2np	The enriched DEGs Gene ID in Gh2WT-vs-Gj2e
Circadian rhythm - plant	CotAD_50720, CotAD_76632, CotAD_49060, CotAD_46528, CotAD_73020, CotAD_55573, CotAD_72073, CotAD_65971, CotAD_33120, CotAD_18936, CotAD_08025, CotAD_57339, CotAD_35151, CotAD_25160, CotAD_61351, CotAD_46346, CotAD_12196, CotAD_74351, CotAD_02653, CotAD_15306, CotAD_32794, CotAD_58236, CotAD_01991, CotAD_14148, CotAD_22354, CotAD_57061, CotAD_72318, CotAD_43458, CotAD_16644, CotAD_69870, CotAD_41425, CotAD_33121, CotAD_63670, CotAD_17426, CotAD_43235, CotAD_34322, CotAD_32839, CotAD_17833, CotAD_11091, CotAD_39072, CotAD_30985, CotAD_10813, CotAD_11502, CotAD_30473	CotAD_06187, CotAD_07275, CotAD_25160, CotAD_38632, CotAD_14148, CotAD_62384, CotAD_32839, CotAD_32848, CotAD_17833, CotAD_47983, CotAD_30985, CotAD_33647
Plant hormone signal transduction	CotAD_40334, CotAD_46528, CotAD_04225, CotAD_28563, CotAD_47590, CotAD_08378, CotAD_49470, CotAD_12194, CotAD_58021, CotAD_06880, CotAD_66459, CotAD_22035, CotAD_31752, CotAD_54679, CotAD_53169, CotAD_45995, CotAD_75512, CotAD_08769, CotAD_18778, CotAD_40223, CotAD_28570, CotAD_73159, CotAD_74752, CotAD_27412, CotAD_38579, CotAD_60775, CotAD_32908, CotAD_38332, CotAD_14880, CotAD_06689, CotAD_15901, CotAD_73761, CotAD_46420, CotAD_29622, CotAD_11634, CotAD_23642, CotAD_60230, CotAD_22796, CotAD_03487, CotAD_15422, CotAD_31462, CotAD_25921, CotAD_44751, CotAD_53340, CotAD_29302, CotAD_49903, CotAD_60363, CotAD_22235, CotAD_48099, CotAD_13396, CotAD_03390, CotAD_19194, CotAD_11283, CotAD_02433, CotAD_33068, CotAD_23314, CotAD_12135, CotAD_28222, CotAD_67654, CotAD_51639, CotAD_12195, CotAD_59532, CotAD_38414, CotAD_54549, CotAD_01401, CotAD_32794, CotAD_08177, CotAD_37030, CotAD_75152, CotAD_57941, CotAD_01991, CotAD_45308, CotAD_08638, CotAD_44201, CotAD_73075, CotAD_63351, CotAD_00235, CotAD_38248, CotAD_01782, CotAD_26378, CotAD_58763, CotAD_07126, CotAD_00351, CotAD_36426, CotAD_11769, CotAD_76883, CotAD_62019, CotAD_62689, CotAD_30952, CotAD_24033, CotAD_50337, CotAD_62680, CotAD_40567, CotAD_72038, CotAD_46354, CotAD_22354, CotAD_67648, CotAD_31477, CotAD_65545, CotAD_65385, CotAD_03246, CotAD_72318, CotAD_17676, CotAD_43458, CotAD_08759, CotAD_18479, CotAD_57227, CotAD_17677, CotAD_59480, CotAD_46553, CotAD_42073, CotAD_08312,	

CotAD_10815,	CotAD_06020,	CotAD_03674,	CotAD_14446,
CotAD_24929,	CotAD_52110,	CotAD_51284,	CotAD_39394,
CotAD_13230,	CotAD_09484,	CotAD_59180,	CotAD_02227,
CotAD_24101,	CotAD_41051,	CotAD_72124,	CotAD_00437,
CotAD_76936,	CotAD_52763,	CotAD_66141,	CotAD_52240,
CotAD_04030,	CotAD_08052,	CotAD_60541,	CotAD_21144,
CotAD_08185,	CotAD_61426,	CotAD_22336,	CotAD_07492,
CotAD_49856,	CotAD_12777,	CotAD_48610,	CotAD_21002,
CotAD_49347,	CotAD_27610,	CotAD_53606,	CotAD_06730,
CotAD_25220,	CotAD_01629,	CotAD_46619,	CotAD_09189,
CotAD_04601,	CotAD_71538,	CotAD_08178,	CotAD_15363,
CotAD_06191,	CotAD_61030,	CotAD_30274,	CotAD_05763,
CotAD_24283,	CotAD_62484,	CotAD_31119,	CotAD_23855,
CotAD_00601,	CotAD_06195,	CotAD_70072,	CotAD_07267,
CotAD_26121,	CotAD_37900,	CotAD_25036,	CotAD_73314,
CotAD_06519,	CotAD_75522,	CotAD_46547,	CotAD_33008,
CotAD_20318,	CotAD_36190,	CotAD_00678,	CotAD_24719,
CotAD_30011,	CotAD_42357,	CotAD_15514,	CotAD_40683,
CotAD_53107,	CotAD_00254,	CotAD_57715,	CotAD_56144,
CotAD_21748,	CotAD_66464,	CotAD_73762,	CotAD_04028,
CotAD_36136,	CotAD_03511,	CotAD_35956,	CotAD_09190,
CotAD_70718,	CotAD_03678,	CotAD_09712,	CotAD_49345,
CotAD_49340,	CotAD_61162,	CotAD_68755,	CotAD_43399,
CotAD_63886,	CotAD_28560,	CotAD_26249,	CotAD_53834,
CotAD_39116,	CotAD_63222,	CotAD_69885,	CotAD_24117,
CotAD_72113,	CotAD_49211,	CotAD_03587,	CotAD_26342,
CotAD_19840,	CotAD_35534,	CotAD_51741,	CotAD_08455,
CotAD_66289,	CotAD_04243,	CotAD_00052,	CotAD_49859,
CotAD_54965,	CotAD_61114,	CotAD_51911,	CotAD_16791,
CotAD_75181,	CotAD_51823,	CotAD_64512,	CotAD_13381,
CotAD_12824,	CotAD_62983,	CotAD_40163,	CotAD_29392,
CotAD_68015,	CotAD_12136,	CotAD_51754,	CotAD_52307,
CotAD_32099,	CotAD_38161,	CotAD_67655,	CotAD_08053,
CotAD_36698,	CotAD_15257,	CotAD_08054,	CotAD_09551,
CotAD_26207,	CotAD_66463,	CotAD_38660,	CotAD_14404,
CotAD_51326,	CotAD_60007,	CotAD_23287,	CotAD_34103,
CotAD_12670,	CotAD_52244,	CotAD_19985,	CotAD_30961,
CotAD_41794,	CotAD_62272,	CotAD_18245,	CotAD_76838,
CotAD_03886,	CotAD_33377,	CotAD_02542	

Supplement Table S8 | The primers list of DEGs used for qRT-PCR

Gene name	Gene ID or Genebank ID	Forward primer	Reverse primer
GhIAA18	CotAD_00235	TTTCTAAAGACACCAAGGAGGAG	GAGGCATCTGGATCAGCAGT
GhBRC1	CotAD_02759	GACATCCGAGACCGAAGTGG	TTTCCCGTGCCTTTACCCTC
GhPCNA2	CotAD_04662	GATGATGAAATGTGCTGGTA	TGATACTCTGCCTCTGGAA
GhPRR5	CotAD_14148	CACATTTACCCTACCACCG	ATCGTCGTTCTTGCTGTC
GhRAV1	CotAD_22031	AAGCAGCATAGATGAAAGCACA	GCGGTAGAGGGAGTCAGGAG
GhEPR1	CotAD_30985	TACCGCCTCCTCGTCCTA	CCCATTGCGTCAGATAAA
GhLHY	CotAD_32839	GATGGAAGCCCAGGAACA	TCGCCCATAGGGTGAAAT
GhSVP	CotAD_47245	CCTCAAAAGGAAGGAAGTAGA	GGTAAGGTAACCCCAGTCTAA
GhBARD1	CotAD_54816	ATCAAGCATCCCTCAGCC	TGTCCCATAGTTCAACCTC
Actin	AY305733	ATCCTCCGTCTTGACCTTG	TGTCCGTCAGGCAACTCAT
UBQ7	DQ116441	GAAGGCATTCCACCTGACCAAC	CTTGACCTTCTTCTTCTTGTGCTTG

Legend of supplementary Data set S1-S13

Supplement Data set S1 | The list of DEGs in compared group Gb1WT versus Gb1np.
Supplement Data set S2 | The list of DEGs in compared group Gb2WT versus Gb2np.
Supplement Data set S3 | The list of DEGs in compared group Gb3WT versus Gb3np.
Supplement Data set S4 | The list of DEGs in compared group Gh1WT versus Gh1np.
Supplement Data set S5 | The list of DEGs in compared group Gh2WT versus Gh2np.
Supplement Data set S6 | The list of DEGs in compared group Gh3WT versus Gh3np.
Supplement Data set S7 | The expression pattern of miRNA
Supplement Data set S8 | The list of DEMs in compared group Gb1WT versus Gb1np.
Supplement Data set S9 | The list of DEMs in compared group Gb2WT versus Gb2np.
Supplement Data set S10 | The list of DEMs in compared group Gb3WT versus Gb3np.
Supplement Data set S11 | The list of DEMs in compared group Gh1WT versus Gh1np.
Supplement Data set S12 | The list of DEMs in compared group Gh2WT versus Gh2np.
Supplement Data set S13 | The list of DEMs in compared group Gh3WT versus Gh3np.
